# The interRAI Suite of Mental Health Assessment Instruments: An Integrated System for the Continuum of Care

**DOI:** 10.3389/fpsyt.2019.00926

**Published:** 2020-01-17

**Authors:** John P. Hirdes, Coline van Everdingen, Jason Ferris, Manuel Franco-Martin, Brant E. Fries, Jyrki Heikkilä, Alice Hirdes, Ron Hoffman, Mary L. James, Lynn Martin, Christopher M. Perlman, Terry Rabinowitz, Shannon L. Stewart, Chantal Van Audenhove

**Affiliations:** ^1^ School of Public Health and Health Systems, University of Waterloo, Waterloo, ON, Canada; ^2^ Psychiatry and Neuropsychology Department, Maastricht University, Maastricht, Netherlands; ^3^ Centre for Health Services Research, Faculty of Medicine, University of Queensland, Brisbane, QLD, Australia; ^4^ Psychiatric Department, Rio Hortega University Hospital, Zamora, Spain; ^5^ Institute of Gerontology, University of Michigan, Ann Arbor, MI, United States; ^6^ Division of Psychiatry, Turku University Hospital, Turku, Finland; ^7^ Graduate Program in Health Promotion, Human Development and Society, Lutheran University of Brazil, Canoas, Brazil; ^8^ School of Criminology and Criminal Justice, Nipissing University, North Bay, ON, Canada; ^9^ Department of Health Sciences for Lynn Martin, Lakehead University, Thunder Bay, ON, Canada; ^10^ Departments of Psychiatry and Family Medicine Larner College of Medicine, University of Vermont, Burlington, VT, United States; ^11^ Faculty of Education, Althouse College, Western University, London, ON, Canada; ^12^ LUCAS Center for Care Research and Consultancy & Academic Center for General Practice in the Department of Public Health and Primary Care, KU Leuven University, Leuven, Belgium

**Keywords:** care planning, outcomes, quality, case-mix, psychometric properties, homelessness, integration

## Abstract

The lives of persons living with mental illness are affected by psychological, biological, social, economic, and environmental factors over the life course. It is therefore unlikely that simple preventive strategies, clinical treatments, therapeutic interventions, or policy options will succeed as singular solutions for the challenges of mental illness. Persons living with mental illness receive services and supports in multiple settings across the health care continuum that are often fragmented, uncoordinated, and inadequately responsive. Appropriate assessment is an important tool that health systems must deploy to respond to the strengths, preferences, and needs of persons with mental illness. However, standard approaches are often focused on measurement of psychiatric symptoms without taking a broader perspective to address issues like growth, development, and aging; physical health and disability; social relationships; economic resources; housing; substance use; involvement with criminal justice; stigma; and recovery. Using conglomerations of instruments to cover more domains is impractical, inconsistent, and incomplete while posing considerable assessment burden. interRAI mental health instruments were developed by a network of over 100 researchers, clinicians, and policy experts from over 35 nations. This includes assessment systems for adults in inpatient psychiatry, community mental health, emergency departments, mobile crisis teams, and long-term care settings, as well as a screening system for police officers. A similar set of instruments is available for child/youth mental health. The instruments form an integrated mental health information system because they share a common assessment language, conceptual basis, clinical emphasis, data collection approach, data elements, and care planning protocols. The key applications of these instruments include care planning, outcome measurement, quality improvement, and resource allocation. The composition of these instruments and psychometric properties are reviewed, and examples related to homeless are used to illustrate the various applications of these assessment systems.

## Introduction

The lives of persons living with mental illness are affected by the interplay of a broad range intrinsic and extrinsic factors emerging over the life course. From the earliest stages of life to the person's final moments, these factors can influence opportunities for growth and development, access to resources, engagement in interpersonal relationships, participation in community, and achieving an overall sense of well-being.

Mental health concerns are both pervasive and heterogeneous. Mental illness and substance use disorders are the world's leading cause of disability (1) accounting for up to 32% of years lived with disability and 13% of disability-adjusted life years (2). Unipolar depression was the fourth leading cause of total disease burden in 2000, after perinatal conditions, lower respiratory infections, and HIV/AIDS (3). With many underlying psychological, biological, social, economic, and environmental causes, it is unlikely that preventive strategies, clinical treatments, therapeutic interventions, or policy options will succeed as singular solutions for the challenges of mental illness.

Persons living with mental illness tend to receive services and supports in multiple settings across the health care continuum that are often fragmented, uncoordinated, and inadequately responsive. Needs may remain unaddressed (4) as the person navigates a path to many providers that function in narrowly defined siloes. Patient flow through episodic service environments (*e.g.*, hospitals) may be constrained (5) and access to appropriate services may be delayed due to lengthy waiting lists (6).

To be effective, any health system's approach must include strategies for identifying and responding to mental illness and related dimensions of health and well-being throughout the life course and in all parts of the health care continuum. As such, the ability for different providers, organizations and sectors to communicate with one another is crucial.

This paper provides an overview of the interRAI suite of mental health instruments, which is designed to function as an integrated assessment and screening system to provide a holistic view of the person's strengths, preferences, and needs. It begins with an examination of the range of factors that must be considered beyond psychiatric symptoms in order to support a person-centered approach to shared decision-making. Next, it describes the design, psychometric properties, and applications of interRAI mental health assessments using examples related to homelessness and trans-institutionalization.

### Assessing the Bigger Picture: The Need to Look Beyond Psychiatric Symptoms

System integration requires the use of a common language for describing needs, monitoring service use, and tracking outcomes over time. Even within a specific care setting, unstructured narrative charts have little value when they are simply “electronic paper records” (7). Medical charts are incomplete, cumbersome, and overly narrow in their focus (8–10). While natural language processing might reduce the burden of reading volumes of narrative notes (11, 12), unstructured charts often have too many information gaps to have value (13). Standardized clinical assessment data can be more useful if they cover the relevant domains, use psychometrically sound data elements, and can be accessed and interpreted for immediate use.

Some clinicians are reluctant to use standardized assessment tools (14) even though their role in evidence-informed practice is recognized (15). Their reasons might include a perceived lack of benefit over clinical judgment, concerns over psychometric properties, and practicality (14). Even if these issues are addressed to one clinician's satisfaction, inconsistency across settings in the choices of measures prevents communication between organizations serving the same individual.

Given the complexity of mental illness over the life course and the likelihood of engaging with diverse service providers, the data requirements for assessment are not rudimentary. Obviously, psychiatric symptoms, cognitive function, diagnosis, and behavioral issues will be highly relevant in most clinical contexts, particularly during acute episodes or mental health crises. However, if the view is widened to the person's broader experience, ability to function, and overall well-being, more domains must be considered as part of a comprehensive assessment of the “whole person”.

#### Life Course Perspective: Growth, Development, and Aging

About 20% of the Canadian population experiences problems with mental health or addictions annually and about half will face mental health problems by age 40 (16). Estimates from New Zealand's Dunedin cohort studies suggest that only 17% of the population will have enduring lifetime freedom from mental illness (17). This is explained, in part, by factors at both ends of life.

About 70% of mental health problems are reported to begin in childhood or adolescence (18), many of which persist over the lifetime (19, 20). Early childhood development sets the stage for social and emotional functioning, academic achievement, and interpersonal relationships (21, 22). As an individual's identity develops in adolescence (23), social networks and peer pressure (24), substance abuse (25), parenting styles (26), and school performance come into play (27). Age-based service restrictions mean the transition from youth to adulthood is often met with fewer mental health resources (28).

Aging is associated with the onset of a number of conditions that can affect mental health, with dementia as the most obvious example (29, 30). Mental illness may accelerate the aging process resulting in shorter life expectancy and greater years of life lost compared with the general population (31–33). Some, but not all, of this is attributable to deaths by suicide (34). Other causes include higher smoking rates leading to increased cancer and cardiovascular disease (35, 36) and higher rates of diabetes arising from poor diet, physical inactivity, and drug related side effects (37). Persons with psychiatric diagnoses who do survive to later life have greater odds of being the most frail (38).

#### Physical Health and Disability

Physical comorbidities are often neglected needs in persons living with mental illness (39, 40). Health care providers are commonly divided according to whether they provide physical health or mental health services. This bifurcation occurs to the detriment of persons living with mental illness who tend to receive lower quality medical care for physical health problems (33, 41). For example, mental health problems are often overlooked or stigmatized in acute hospital settings (33), cancer screening tends to be inadequate (42), and effective therapeutic regimens are less likely to be received by patients with mental illness who have acute myocardial infarction (43). In mental health settings, treatment of physical health problems is often delayed or inadequate (44, 45), and health promotion may be regarded as a low priority (46).

Mental health issues may be both consequences and causes of physical disability. A four-nation study showed that impaired physical functioning and dual sensory loss were associated with greater odds of depressive symptoms in home care clients (47). Suicide attempts may cause disability among survivors, but also there is increased risk of suicide among persons living with physical disabilities (48–51) for reasons that include pain and a sense of burden to others (52, 53).

#### Social Relationships

Social isolation and loneliness are increasingly recognized as important risk factors for the physical and mental health of persons of all ages (54–56). For example, social support networks may play an important role in facilitating recovery from mental health problems (57) and they may provide instrumental support with tasks such as child care, transportation, or medical management (58). On the other hand, mental health problems may pose barriers to forming or maintaining close personal relationships over the life course (59, 60). In addition, social relationships may be a source of stress, trauma, or abuse (61–63) that can have immediate and long-term consequences for mental health. Hence, interventions must consider both the social resources that may be beneficial and relationships that may be detrimental to the person's well-being.

#### Economic Resources

The pathways between poverty, deprivation and mental illness are multifold. Low income households are associated with higher rates of depression and antisocial behavior in children, but the impact may lessen if household resources improve (64). Job loss may pose tremendous stress on individuals and families resulting in worsened physical and mental health (65). However, for persons with pre-existing mental illness, unemployment and poverty are pervasive (66) resulting in reduced access to adequate food, shelter, clothing, and medical treatment. The stigma associated with poverty and mental illness (67, 68) may be further magnified in low resource nations (69).

#### Homelessness and Housing

Stable housing is an important prerequisite for recovery. Persons who lack stable housing, particularly the homeless, commonly experience higher rates of physical and mental illness, poor quality of life and high mortality (70–74). At the core of homelessness are the processes of marginalization and exclusion, with multiple risk factors driving these experiences (75–78). For instance, persons with childhood trauma, mental illness, and substance use are at greater risk for housing instability and homelessness (79). Homelessness arises from an interplay of individual vulnerabilities, interpersonal, structural, and systemic factors. Individual factors consist of psychosocial vulnerabilities connected to family background, health, and trauma. Interpersonal factors are linked to behaviors like substance use. Poverty, unemployment, and lack of affordable housing are examples of the structural factors; lack of social security of the systemic ones.

#### Substance Use

The relationship between substance use and mental illness is multifaceted. Based on a recent meta-analysis, more than half of persons with mental illness have a comorbid substance use disorder (80). This comorbidity leads to poorer clinical outcomes, worsened physical and mental health; higher levels of disability; increased risk of suicidal behavior (81), homelessness, and psychiatric readmissions (82); and greater difficulties in interpersonal and family relationships (80). Symptoms of mental illness may interfere with substance use treatment and the reverse may also be true (83).

Proposed mechanisms for this comorbidity include self-medication where people use substances to treat their own mental health symptoms; the reverse-causal pathway where substance use causes or worsens the symptoms of mental illness or side effects of substances produce symptoms similar to mental illness; and a shared environmental or genetic vulnerability where factors like poverty or childhood trauma increase risk for both (84). Considering the complexity of these relationships, comprehensive assessment is needed to understand the context of substance use in relation to symptoms of mental illness.

#### Trans-Institutionalization and the Criminal Justice System

Deinstitutionalization of mental health services began in Canada and other countries in the 1960s–70s with the rapid closure of psychiatric hospitals followed by further reductions in general hospital admissions for mental health in the 1990s (85). In most countries, the motivations for this change included breakthroughs in pharmaceutical treatments (86) combined with an emphasis on human rights and social inclusion of persons with mental illness (87), and the expectation of better outcomes in the community (88). However, legal concerns and fiscal decisions aimed at reducing health care expenditures (85, 89) were also important drivers. Globally, there is considerable variability in rates of deinstitutionalization (90, 91) despite broad based philosophical support (92, 93).

Deinstitutionalization is associated with improved life expectancy for persons with mental disorders (94), disability reduction (90, 91), better quality of life, and greater autonomy (95). Adverse consequences include a reported rise in trans-institutionalization, homelessness, and criminalization of persons with mental illness (96–99). While there is debate about the extent to which deinstitutionalization has resulted in negative outcomes (100, 101), contact with the criminal justice system is now a common experience for persons with mental illness.

Police have become the first line of contact for many persons living with mental illness (102, 103), with substance use and impulse control issues as common causes (104). In some jurisdictions, the large-scale expansion of inpatient forensic mental health services has been driven by increased admissions of younger individuals from ethno-racial minorities with low levels of violence and substance use offences (105). Specialized mental health courts provide alternative mechanisms for dealing with offenders with mental illness (106), and more advanced analytics of data based on high quality assessments will be essential for the improvement of forensic mental health services (107).

#### Recovery

The shift to community-based mental health services has been accompanied by a transition from a biomedical care model to one that emphasizes personal recovery (108–110). While there is no common global definition of recovery (111), there are agreed-upon common core principles. For example, that mental health is viewed in terms of a continuum of functioning; recovery may be an iterative or non-linear process; and belonging, community engagement, hope, connectedness, identity, freedom from stigma, meaning in life, and empowerment are key markers to consider (108). Providing a supportive environment that builds on strengths and is calibrated to needs is a priority, rather than stopping services (112). Treatment of symptoms is important, but “clinical recovery” is either not enough or not fully possible in some cases. Therefore, assessment must consider the person in a broader social context to reach beyond symptoms alone.

#### Stigma

Stigma associated with mental health problems often hinders full citizenship and inclusion in society (113, 114). At the societal level, *public stigma* includes labeling persons with mental illness as dangerous or less competent than the general population. At the individual level, *self-stigma* occurs when persons apply psychiatric labels to their identity, often with negative consequences. Those who experience perceived stigma or discrimination may apply stereotypes to themselves resulting in underestimation of their own potential and a sense of helplessness (115). This may lead to avoidance of necessary care, increasing problems in multiple domains, and worsened prognosis (116, 117).

### Conglomeration *Versus* Integration of Assessment

If we accept that standardized assessment has value, we must consider how to define that standard. In many health care settings, usual practice has been to create a conglomerated information system made up of home-grown intake forms combined with a collection of clinical scales used to measure singular issues (*e.g.*, cognition, depression) on an “as-needed” basis.

Data from these conglomerated systems are typically not automated and forms completed separately are not linked. For the service recipient, this can lead to repetitive measurement of the same issues and limited access to clinically relevant information for team members in the circle of care. Longitudinal case histories cannot be constructed easily, linkages between clinical issues tracked on different forms may go unrecognized, and person-level analytics cannot be readily applied to the clinical record. Data compiled this way have little utility for population level analyses because converting them into a complete, electronic database is a daunting task. Handwritten notes on incomplete forms stored in locked filing cabinets serve little purpose beyond single-case after-the-fact chart audits.

Even if data are automated, when the choice of which scale to use for a particular concern is left to individual clinicians, there will almost certainly be no measurement consistency at the organization or system levels. *The Handbook of Psychiatric Measures* (118) contains 26 chapters with over 275 distinct psychiatric measures, and that list was almost surely incomplete at the time of publication. Assuming one could resolve the controversy over whether the Montreal Cognitive Assessment should be used in place of the Mini Mental State Examination (119), what is the likelihood of a consensus on which of the nine depression measures listed in the *Handbook* should be used with all patients, in all settings, during all episodes of care? At the population level, inconsistent data have diminished value. Imagine the response by an organization ranked negatively in an outcome-based quality indicator where different providers use different tools. The fact that an indicator was “not measured the same way” across settings will be the immediate defense used to negate the validity of any differences in performance suggested by the indicator.

Using specific scales on an “as-needed” basis is also a flawed strategy. We do not always know who has a given problem without asking. Indeed, one of the values of comprehensive assessments is that they force the clinicians to ask about underlying issues that go beyond the immediate, presumed problem. Persons with substance use problems are not only found in substance use programs; depression affects people other than those in a mood disorder program; cognitive impairment spans the barriers of a dementia unit. Under-detection of issues like pain, depression and dyspnea is an important problem throughout the health care system (120–122).

If we succeeded in achieving a consensus on a set of tools to measure psychiatric symptoms using a collection of single-focus measures, the data volume and time to complete assessment could still be unwieldy. For example, a set of 15 well-known mental health instruments would measure cognition, (123), delirium (124), depression (125), anxiety (126), psychosis (127), mania (128), trauma (129), pain (130), sleep (131), behavior (132), substance use (133), instrumental activities of daily living (134), functional status (135), suicidality (136), and aggression (137). Despite comprising more than 400 different items, this set does not address many of the key issues highlighted previously.

Further, intellectual property and licensing requirements may be difficult to resolve. Within a single clinical setting the task of securing permissions for 15 or more different instruments would be a challenge, but to do so on a national level is impractical. This would be further complicated by electronic health record vendors licensing requirements, as well as the challenge of training clinical staff on coding conventions that likely differ dramatically among instruments. Finally, though the conglomerated set of assessments may measure the severity of an issue at a given time and can be used on a repeated basis to monitor changes, these measures generally do not invoke a clinical response in reaction to their numeric scores.

The alternative to a conglomeration of stand-alone tools is to use a single, integrated assessment system that can be applied across care settings to persons of any age. The system should serve multiple applications for multiple audiences, including triggering a clinical response leading to shared decision-making in support of key recovery goals.

## The interRAI Suite of Mental Health Instruments: An Integrated Assessment System

interRAI (www.interRAI.org) is a not-for-profit network of over 100 researchers, clinicians and policy experts from over 35 countries in North and South America, Europe, Asia, Africa and Oceania (138–140). It was founded with an initial focus on geriatric research in the early 1990s, but its scope broadened to include vulnerable persons of all ages. interRAI's multinational program of research aims to develop and implement comprehensive assessment and screening systems to support improved quality of care and quality of life among persons of all ages with complex needs across the continuum of health and social service settings. This includes the creation of psychometrically sound measurement systems, the application of data from those assessments to support multiple uses by multiple audiences, and completion of multinational comparative analyses and “natural policy experiments.”

The initial innovation behind interRAI assessments is that they were designed not only to describe status or severity at a given time, but also to invoke a clinical response through the use of embedded triggering algorithms and care planning guidelines (141).

The first interRAI instruments were designed to be used in a single sector at a time (142, 143), and the release of interRAI's mental health instrument for in-patient psychiatry (144) represented its first effort to target the general adult population. In 2000, interRAI launched a major effort to redesign all of its assessment instruments to function as a fully integrated suite of measures (145–148). The most recent developments by interRAI include the creation of a parallel suite of instruments for children and youth (149, 150), screening systems for use by non-health professionals (151), and a set of self-report tools to measure patient experience (152–154) and patient reported outcome measures(155, 156).

The interRAI suite of assessment instruments includes over 20 comprehensive assessments, supplementary assessments, and screeners. All of these systems include indicators related to mental health (particularly cognition and depressive symptoms). However, the focus in the following section will be on the adult versions of the mental health instruments using homelessness to illustrate how they can provide insights about strengths, preferences, and needs.

### Assessment and Screening Instruments for Adults

#### Mental Health Settings

##### Inpatient Psychiatry

The interRAI Mental Health (MH) assessment (157, 158) supports care plan development in 20 domains, and is used in in-patient mental health settings at admission, discharge (if more than 7 days after admission), every 90 days (for long-stay patients), and when there is a clinically significant change in the person's status that is not a self-limiting and could require modifications to the care plan. The instrument is available in English, Canadian and Belgian French, Flemish, Icelandic, and Finnish.

The first version of the instrument was released in 1999, with a major update in 2002. The most recent version 9.1.2 was published in 2012 (157) with revisions designed to make it fully compatible with the interRAI suite (147). The MH was pilot tested in Nordic countries and the US, but the primary implementation has been in two Canadian provinces (Ontario, Newfoundland and Labrador). Local implementations have occurred in two other provinces (Quebec, Manitoba). To date over 1.4 million assessments have been completed on over 320,000 unique individuals in Canada.

The target audience is adults aged 18 years and older, including acute, long-stay, forensic and geriatric patients. Canadian provinces have also implemented the MH in adult-designated beds, even if those are occupied by persons under 18 years of age. This proved to be a helpful source of data for publications that pre-dated the completion of the child-youth suite of mental health instruments (159).

The assessment is comprised of 396 items in several domains (see **Table 1**). It is mainly completed by mental health professionals (typically nurses or social workers), and includes a limited number of self-report items. Most items employ a standard 3-day look-back period, although service use and therapies use a standard 7-day window. Other items use 30-day, 90-day or lifetime estimates depending on the nature of the issue. The average time to complete the assessment is 1 hour, and it can be used as a replacement for a standard nursing intake assessment. The MH instrument deals with items that usually would be addressed in mental health assessments, but also expands the scope to look at broader issues within a time frame for completion that is consistent with conventional practice. It contains about the same number of items as the hypothetical conglomerate of stand-alone tools described earlier, but it substantially increases the scope of issues addressed.

**Table 1:** Item counts by domain area and interRAI mental health system for adult populations (age 18+ years).

Characteristic	interRAI Assessment or Screening Instrument					
	Mental Health (MH)	Community Mental Health (CMH)	Emergency Screener for Psychiatry (ESP)	Brief Mental Health Screener (BMHS)	Forensic Supplement (FS)	Addiction Supplement (AS)
Setting	Inpatient psychiatry	Community (ACT, case management)	Emergency department, mobile crisis	Police, EMS, other settings without MH staff	Forensic services	Addiction programs
***Item counts***						
Administrative & tracking	43	39	27	22	7	6
Mental status indicators	42	40	33	8	6	0
Substance use/addictions	17	19	10	1	0	17
Harm to self/others	13	17	12	9	11	0
Behavior	9	6	5	2	3	0
Cognition	8	8	5	1	0	0
Functional status	16	23	3	0	0	0
Communication & vision	4	4	1	0	0	0
Physical health conditions	40	41	0	0	0	11
Stress & trauma	19	21	1	0	1	0
Medications	5^1^	4^1^	4	1	0	0
Service use & treatments	36	36	2	0	0	1
Control interventions	13	0	0	0	0	0
Nutritional status	10	10	0	0	0	0
Social relations	12	31	9	0	5	1
Employment, education & finances	9	9	0	0	0	0
Housing, Home environment, Living arrangements	5	10	6^2^	3^2^	0	0
Diagnoses	30	28^3^	16	0	0	0

^1^An additional detailed list of medications used in the last 3 days is optional. ^2^Home environment assessed only if home visited by staff or key informants. ^3^Section allows for entry of additional DSM/ICD diagnoses as needed.

##### Community Mental Health

The interRAI Community Mental Health (CMH) assessment (160) supports care plan development in 20 domains. It is designed for community agencies employing mental health clinicians, including those with case managed mental health services and assertive community treatment programs. The assessment can be used at intake, discharge, every 6 months depending on length of stay, and after a change in the person's status that requires care plan modification. If the person is admitted to the community agency from an inpatient setting using the MH, the discharge MH assessment is shared to support continuity of care (161) to allow additional time for community staff to establish a clinical relationship with the person. The target audience for the CMH is also adults aged 18 years and older, including a broad range of service recipients. Although community mental health agencies are the typical service provider, it has been used in consultation liaison services for long-term care to deal with psychiatric and behavioral issues outside the usual scope of practice for clinicians in those settings as well as a Dutch study of homeless services recipients (162). The original version of the CMH released in 2007 was pilot tested in Ontario, Canada, New York State, Finland, Belgium, Chile and Hong Kong. The most current version 9.2 published in 2012 is compatible with the interRAI suite. Newfoundland and Labrador have begun provincial implementation of the system, as well as regions in Ontario and Quebec. The Swiss Home Care association has announced plans for national implementation. The instrument is available in English, French (Canadian, Belgian, Swiss), Swiss German, Swiss Italian, Flemish, Finnish, and Chinese. For this paper, 12,862 assessments from pilot studies are available for analyses. Because of constraints related to European data protection laws, it was not possible to complete pooled analyses with those data (162).

The CMH includes 405 items dealing with comparable domains and look-back periods as used in the MH. The two instruments share 330 common items, but the CMH has 75 items not present in the MH and the MH has 66 items not present in the CMH. The main differences relate to issues that are encountered in one, but not the other, service environment. For example, the CMH includes an expanded range of items on social relationships and supports in the community, and on the home environment. The MH includes items on control interventions (*e.g.*, restraints) not used in community settings.

The average time to complete the CMH is also an hour, but completion time may be affected by the lack of access to another informant for persons who have difficulty responding. This issue poses a greater challenge than noted in the interRAI Home Care assessment (163) where caregivers are routinely available as additional sources of information.

##### Emergency and Crisis Services

The interRAI Emergency Screener for Psychiatry (ESP) (164) is designed for general emergency departments, psychiatric emergency departments, and mobile crisis teams. Like the MH and CMH, the ESP is typically completed by nurses, social workers, or clinicians other than psychiatrists. It is used at the time of crisis/emergency, with the expectation that a follow-up assessment for persons remaining on service would revert to the MH or CMH once the person is stabilized.

The first version of the ESP was pilot tested in Ontario in 2004, and the interRAI suite version 9.1 was published in 2011. The ESP's 141 items are a subset of those in the MH and CMH. The instrument has been pilot tested in Ontario and Quebec, Canada and Finland. Two regional mental health services in Canada have begun implementation of the ESP and a child-youth variant of the instrument. The current data holdings available at the time of writing comprised 5,249 ESPs completed in Canadian organizations.

The target audience for the ESP is also adults aged 18 years and older; however, the clinical focus of the ESP is narrower. Whereas the MH and CMH focus on care plan development in 20 areas, the emphasis of the ESP is on patient safety issues and acute symptoms. Thus, the ESP has a 24-hour look back period to focus on immediate presenting concerns. It triggers care plans in three areas of safety (harm to self, harm to others, inability to care for self), and in substance related withdrawal symptoms.

The average time to complete the ESP is 30 min; however, the acute nature of illness may pose barriers to continuous completion of the assessment if other informants are not available.

##### Police, Emergency Medical Services, and Other Non-Mental Health Settings

The interRAI Brief Mental Health Screener (BMHS) is a short screening tool intended to be used by non-mental health professionals who may be the first line of contact for persons with mental health needs (165). The initial target audience for the BMHS is police services (151) with the aim of facilitating improved communications among polices officers, emergency mental health services, and community mental health agencies (166). The BMHS is a 46-item subset of the ESP, but the training manual includes additional materials for training non-mental health staff in the use of appropriate terminology to describe the person's presenting symptoms. It is designed to be used at a single time point, and it employs a 24-hour look back period.

The first draft version of the BMHS was developed using analyses of 41,019 MH admission assessments to determine the minimum subset of items needed to identify persons who would be admitted to inpatient psychiatry due a combination of disordered thought and danger to public safety (167). The draft BMHS was tested with two police services and five hospitals in Ontario in 2011. The finalized version 9.3 of the BMHS was published in English and French in 2015, and the screener is now in extensive use by over 40 police agencies (local, provincial, and federal) in four Canadian provinces. A pilot study of the BMHS in one US region is expected to launch in 2020. For the purposes of the present paper, 70,005 Canadian BMHS assessments were used.

Unlike the previous assessments, the BMHS is not a care planning tool. It is designed to provide a systematic means of summarizing the observations of police officers (and other non-mental health staff) using the same items that are employed in the interRAI mental health suite. It can also be considered a mental health training intervention, because the screener provides guidance to those using the instrument on how to identify and describe mental health symptoms. The BMHS includes three patient safety algorithms related to harm to self, harm to others, and inability to care for self based on machine learning analyses of MH and BMHS data (168). These algorithms help police and others to communicate acuity to hospitals and community mental health agencies in real time. The Ontario Human Services and Justice Coordinating Committee recommended the province-wide use of the BMHS in 2019 in order to facilitate more timely and appropriate transfers of the care of persons with mental illness from police control to mental health agencies (169).

There is considerable potential to employ the BMHS in other settings. Emergency Medical Services are also in frequent contact with persons with mental health crises in the community and face similar decision-making challenges. In addition, a non-police version of the BMHS is available that could be used with peer-led agencies, shelters, food banks, or other settings for persons with mental illness.

##### Forensic and Addictions Services

The interRAI MH and CMH instruments both address a broad range of adult service recipients, including persons with addictions and those in contact with the criminal justice system (170–172). However, in forensic and addictions programs, there may be value in obtaining additional information about the severity of the problem (*e.g.*, criminal convictions) or items that would not be asked routinely for all service recipients (*e.g.*, readiness for change, health symptoms associated with substance abuse, dynamic and static forensic risk factors).

To that end, interRAI has two new supplements for the MH and CMH to expand depth in criminal justice and addictions with 33- and 35-items, respectively. These items provide additional severity measures, risk algorithms and specialized care planning triggers for these clinical subpopulations only. At the time of writing, both supplements are in beta versions, with expected publication in 2020.

#### Non-Mental Health Settings for Adults

All interRAI assessment and screening systems, including those for non-mental health settings, have at least a core set of mental health items. A brief description of some of the main adult instruments follows with examples of mental health research done in those settings.

##### Intellectual Disability

The interRAI Intellectual Disability (ID) assessment (173, 174) is used with adults aged 18 years and older with intellectual and developmental disabilities (*e.g.*, Down syndrome, autism) in community and residential services. It comprises 320 items, including 188 in common with the interRAI MH, and it is usually completed by developmental services workers. The ID includes support planning protocols for abuse by others, communication, continence, injurious behavior, meaningful activities, mental illness, and social relationships (175). The mental health content of the ID includes measures of psychological well-being, stressful life events and trauma, cognition, psychosis, extrapyramidal symptoms, mood, anxiety, negative symptoms, sleep disturbance, behaviors, violence, and previous psychiatric hospitalizations. These indicators have been the focus of a number of studies in ID settings (176–180) as well as cross-national comparison of persons with ID (181).

The interRAI ID has been implemented in the state of New York (USA) and in Prince Edward Island (Canada). It was used in Ontario, Canada to support the closure of the province's last three large institutions for persons with intellectual disabilities (179, 180, 182–184). Several other jurisdictions in the USA and Canada have announced plans to implement the ID. ID supplements to the MH and CMH instruments are currently being pilot tested in Ontario and in Belgium.

All interRAI mental health assessments include items on intellectual disability, since persons with dual psychiatric and ID diagnoses are an important subpopulation in mental health settings. Several interRAI papers have examined persons with intellectual disabilities in trans-institutional settings (179, 185–189).

##### Home Care and Nursing Homes

interRAI's Long Term Care Facility (LTCF) and Home Care (HC) instruments (142, 143, 163, 190, 191) were first developed more than 25 year ago. By the end of 2018, over 9 million interRAI home care and nursing home assessments had been completed in Canada alone. In the US, since 1990, over 100 million interRAI nursing home assessments have been completed. The use of LTCF and HC is worldwide; other nations with large scale implementations of one or both of these instruments include Iceland, Finland, Belgium, France, Switzerland, Italy, Hong Kong, and New Zealand.

The LTCF and HC contain 322 and 318 items, respectively, and are completed by health professionals (typically nurses or social workers). The mental health items assessed in both instruments include cognition, delirium, mood, behaviors, psychosocial well-being, psychosis, alcohol and psychotropic medications. The 27 care planning protocols triggered by the HC and LTCF include ones dealing with cognitive loss, delirium, mood, behavior, abusive relationships, tobacco and alcohol use, and appropriate medications (192).

The mental health research in long-term care with the LTCF and HC includes depression (193), bipolar disorder (194, 195), suicidal behaviors (196, 197), traumatic brain injury (198–200), delirium(201, 202), aggressive behavior (203–205), and cognitive impairment (206–210).

Two quality of care issues of considerable interest are use of physical restraints (211–218) and potentially inappropriate use of antipsychotics (211–214, 219–225). The Canadian Institute for Health Information (CIHI) now reports interRAI's risk adjusted quality indicators for restraint and antipsychotic use in nursing homes in seven provinces/territories. This will expand to all other jurisdictions except Quebec once current implementations are complete.

#### Instruments for Children and Youth

The newest instruments in the interRAI mental health suite are those designed for children and youth. Implementation of the interRAI MH in all adult inpatient beds in Ontario was mandated in 2005 in response to recommendations from the province's Joint Policy and Planning Committee (JPPC) of the Ministry of Health and Long Term Care and the Ontario Hospital Association. The JPPC also called for development and implementation of a compatible system for children and youth. In response, interRAI researchers developed the interRAI Child/Youth Mental Health (ChYMH) assessment system for children aged 4–18 in mental health settings (226) as well as a shorter screener (227).

The ChYMH has 382 items and 31 summary scales (*e.g.*, internalizing, externalizing, distractibility and hyperactivity, aggression, anxiety, social disengagement, depression severity index), and risk assessments to inform the intensity and nature of the child or youth's service needs (149, 159, 228–236). Additionally, the ChYMH has 30 care planning protocols and a preliminary system to describe resource intensity (237). An Adolescent Supplement covers items not addressed with younger children (*e.g.*, substance use) and a Youth Justice Supplement is for youth in correctional settings. A variant of the ChYMH is also available for children/youth with intellectual and developmental disabilities (238). Finally, a new instrument for newborns to three-year olds is nearing completion (239).

The interRAI ChYMH has already been adopted by 90 children's agencies in Ontario, and three other provinces have expressed interests in adopting the system. In addition, there were 16,588 MH assessments on children aged 13–17 in adult mental health beds in Ontario by December 2018.

The availability of an integrated suite of mental health assessment systems that span the life course from newborns to centenarians provides enormous potential to improve care with various transitions that occur over the life span. In addition, at the person-level these compatible assessment systems can provide a rich continuous clinical picture of the person's growth, development and aging from a comprehensive perspective. At the population level, the large-scale implementations of these instruments portend the emergence of a new, detailed longitudinal database of large cohorts of individuals living with mental illness in the earliest stages of their lives until the latest stages and end of life.

### What Makes interRAI an Integrated Mental Health Information System?

The following factors make interRAI's mental health assessments and screeners an integrated health information system that spans the continuum of care for persons of all ages. In this and subsequent sections, interRAI data holdings are used to illustrate concepts discussed below. **Table 2** provides a summary of the data sources used in these examples.

**Table 2:** Data sources used to illustrate concepts in manuscript.

Instrument	Country	Setting	Type of implementation	Base sample N^4^	Population level data?	Notes
Mental Health (MH)	Canada (NL, ON, MB)	Psychiatric Hospitals/units	Mandated use	230,790	Yes	Unique individuals. Most recent episode 2005–17. Excludes stays <3 days.
Community Mental Health (CMH)	Canada (NL, ON), USA, Finland Netherlands	Community mental health Homeless services	Pilot sites & early adopters Research sites	CA-8,627; US-2,689; FI-1,506 436	No No	Unique individuals. First assessments 2007–17.
Emergency Screener for Psychiatry (ESP)	Canada (ON)	Emergency rooms, mobile crisis teams	Pilot sites, early adopters	5,264	No	Assessments between 2007 and 17
Brief Mental Health Screener (BMHS)	Canada (ON, MB, SK)	Police services	Early adopters	70,005	Yes	Screeners between 2014 and 18
Long-term Care Facility (LTCF)	Canada (NL, NS, ON, MB, SK, AB, BC, YT) Canada (ON, MB)	Nursing homes Complex Cont'g Care hospitals	Mandated use Mandated use	470,350 268,685	Yes Yes	Unique individuals. Most recent episode, 2010–18 Unique individuals. Most recent episode, 1996–2018
Home Care (HC)	Canada (NL, NS, ON, MB, SK, AB, BC, YT)	Home care agencies	Mandated use	718,721	Yes	Unique individuals. First episode 2003–18
Community Health (CHA)	Canada (ON)	Community support services	Mandated use	28,965	Partial	Unique individuals. First episode 2012–18
Palliative Care (PC)	Canada (ON)	Community palliative care	Mandated use	40,013	Yes	Unique individuals. Assessments from first episode 2011–18

^4^N's in some tables vary because they use subsets of the base sample.

#### Common Language

All interRAI instruments use common terminology to define the same items across all settings. Mood, psychosis, cognition, pain, function and physical health systems use the same definitions, inclusion/exclusion criteria, and phrasing wherever they appear irrespective of the type of setting. Items in the child/youth instruments only differ from the adult instruments when a developmental rationale requires the difference (*e.g.*, performance of activities of daily living). Also, most instruments employ a standard 3-day look back period; except the ESP and BMHS use a 24-h look back due to the acute nature of the conditions addressed.

#### Common Conceptual Basis

All interRAI assessment instruments are designed first and foremost to support care planning, by embedding data elements where algorithms trigger the need for further intervention in a given area. The development process for instruments always begins with identifying the focal domains for care planning and then identifying the minimum item set to trigger the need for additional investigation. Secondary consideration is given to items that are not used for care planning, but could be used for outcome measurement, resource allocation, of quality management. Specific items to only track matters of research interest are not included in these systems.

#### Common Clinical Emphasis

The primary focus of interRAI assessments is on function and symptoms rather than diagnosis. These instruments track provisional and finalized psychiatric and medical diagnoses and they provide rich clinical information that is relevant to diagnosis, but they are not intended to be diagnostic systems. Rather they focus on what are the person's strengths and needs, how the person relates to others and engages in the community, what the person can do independently, and where support needs are required.

#### Common Data Collection Methods

All interRAI systems employ a common and consistent assessment methodology. For the clinician-administered instruments, the assessor is provided with specific item definitions, time frames, inclusion/exclusion criteria, lists of examples, coding guidelines, and instructions for the assessment approach. The assessments do not use a fixed narrative set of questions, and the order of completion can be adapted to the natural flow of the assessment process on a case-by-case basis. Assessors are aware of the information they need to acquire for a given item, but they are free to adapt approaches to each item to be culturally or gender-appropriate. Assessors use all sources of information available to them, including direct questions posed to the person, observation of the person's behavior and mannerisms, interviews with family or other members of the support network (where appropriate), information exchanges with other members of the circle of care, and medical or other records. When these information sources provide conflicting evidence, clinicians exercise their best judgement to record what response they believe to be the most correct answer for a given item. It is also possible to parcel out portions of the assessment to different staff members. For example, clerical staff could readily complete administrative and tracking elements or data on historical service utilization. Other mental health disciplines may complete some but not all portions of the assessment. For auditing practice, one individual must sign-off that the assessment is complete and as accurate as possible given available information.

This assessment approach is standardized for all interRAI systems and shared items are assessed in the same manner. What differs among instruments is the curated item sets and associated scales and algorithms. Thus, information between sectors can be compared directly, and staff with experience in interRAI systems in one sector can easily learn another interRAI instrument if they move to another sector. While items sets and clinical issues may be new, the approach to completing items remains consistent.

#### Common Core Elements

Mood, pain, cognition, and functional status are basic human characteristics that are relevant in any setting and any age group. In that light, there is a set of common items that are measured in almost all instruments. The main exceptions are screeners that are intended to use a reduced item set for limited targeting purposes, and the newborn to three year-old instrument that has items dealing with earliest stages of human development.

**Figure 1:**
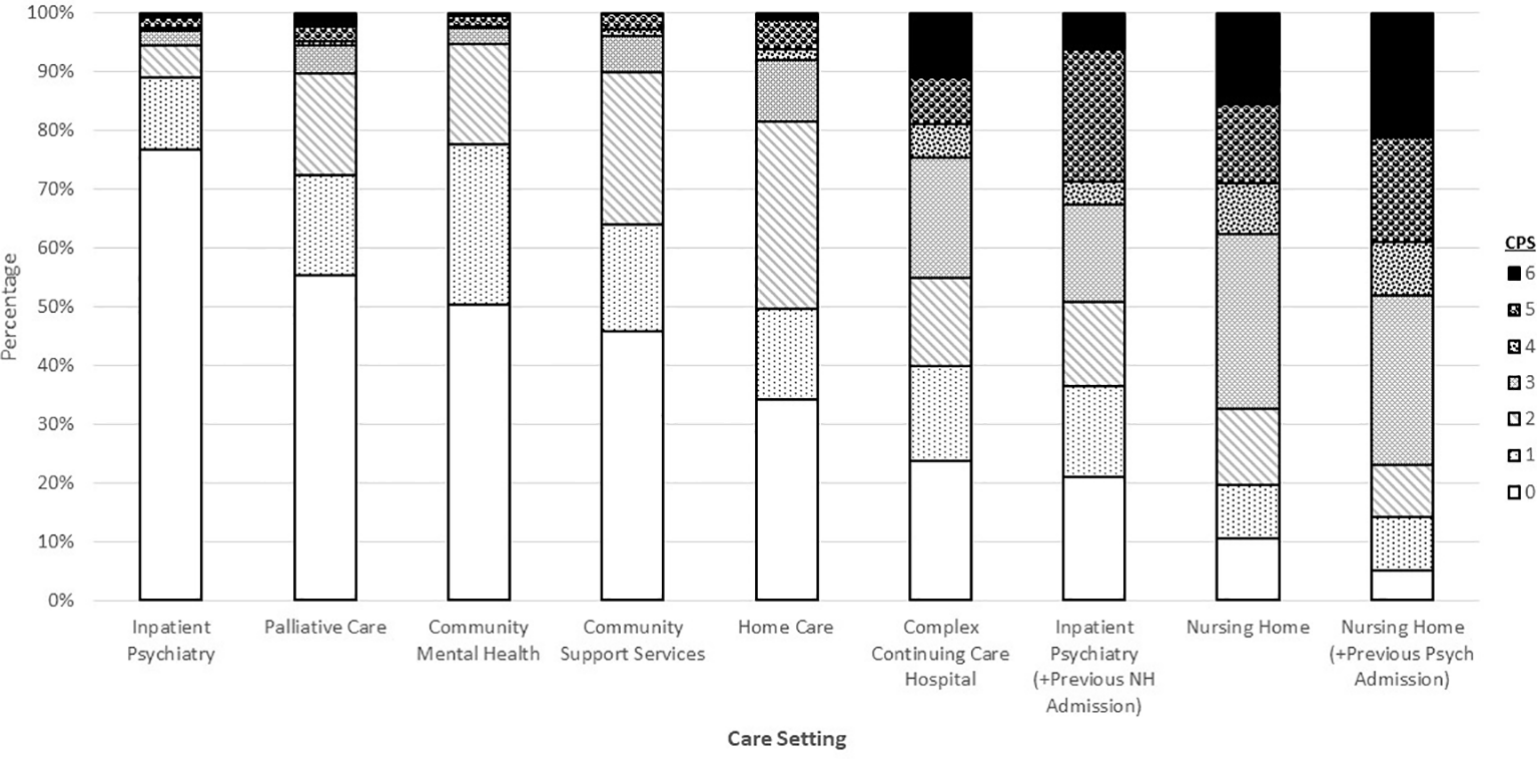
Percentage distribution of cognitive performance scale scores across Canadian care settings.

This consistency of measurement allows for the examination of some clinical issues at the population level. For example, **Figure 1** shows the distribution of interRAI's Cognitive Performance Scale for about 1.7 million individuals across a variety of adult service settings in Canada (see **Table 2** for data sources). Scores range from zero for cognitively intact to six for very severely cognitively impaired. There is a clear transition from less to greater cognitive impairment as one moves from populations whose usual residence is in the community to facility-based settings. The most severely cognitive impaired population is nursing home residents with a previous history of psychiatric hospital admissions. The third most severely impaired population is inpatient psychiatry patients who were admitted from nursing homes. Both of these trans-institutional populations have much more severe levels of cognitive impairment than the general inpatient psychiatry population.

#### Common Care Planning Protocols

interRAI's clinical assessment protocols are designed to inform the care planning process for issues identified in the assessment. These protocols are grouped according to clusters of care settings serving persons with common clinical issues who may nonetheless be at different points in the treatment trajectory. The MH, CMH, and ESP are adjacent sectors of the mental health system that use the same set of mental health protocols to address the needs of their shared populations. Similarly, the LTCF, HC and interRAI Community Health Assessment share common protocols for a population that is generally affected by geriatric issues or issues related to physical disability paired with medical complexity and/or cognitive impairment. The protocols for supporting adults with ID can be used in residential and community settings, and the ChYMH instruments have shared protocols for community and facility based settings for children/youth.

### Psychometric Properties of interRAI Mental Health Instruments

One of the main value propositions for adopting research-based assessment systems over “home grown” bespoke forms is that decision-makers must have confidence in the veracity of the findings generated by the assessment. The data must be meaningful, accurate, and relevant to the issues affecting the person who has been assessed. Reliability and validity are two basic psychometric properties to be considered and the cornerstone for instrument development (240).

#### Reliability

##### Inter-Rater Reliability

In any health care system it is essential that independent assessments by different trained clinicians yield consistent answers. To be cost-effective and to minimize assessment burden, it should be feasible to use assessment information completed by another colleague (including from a different care setting) to inform our own clinical decision-making process.

To appraise this, interRAI has a long history of engaging in inter-rater reliability testing of its assessment systems (142, 143, 191, 241–248). The approach used has been to conduct independent assessments of service recipients without contact between the assessors and with a limited time gap between assessments. This conservative, yet pragmatic, approach mimics what may occur in usual practice in health care settings. Although it is convenient to use vignettes in reliability research they are avoided because they do not capture the nuances and complexities of “real life”. Similarly, assessors do not work together in pairs because that approach violates assumptions of independence between observers (249), which could inflate reliability estimates and does not reflect usual practice patterns in health care.

Recognizing that interRAI assessments share a common core of data elements that are combined with specialized items unique to a given sector, it is feasible to use inter-rater reliability evidence from multiple sectors to evaluate instrument performance. In a 12-nation study of inter-rater reliability of five different interRAI instruments (MH, LTCF, HC, Palliative Care, and interRAI Post-Acute Care), the mean weighted kappa for the core items common to all instruments was 0.75 and the kappa of the specialized mental health items was 0.64 (145). Both results suggest “substantial” inter-rater reliability (250).

##### Internal Consistency

Challenges with inter-rater reliability studies are that they are expensive, intrusive to the person being assessed twice, and of no clinical benefit to that person. Therefore, it is useful to employ other statistical approaches to reliability evaluation that can be done continuously without additional assessment. When instruments use parallel form scales to measure underlying domains with multiple items, it is possible to compute measures of internal consistency using Cronbach's alpha (251). This approach is used to evaluate new scales developed for the interRAI suite, but it can also be deployed to monitor data quality continuously with large scale implementations as has been done with other interRAI assessments (252, 253).

**Table 3** describes several clinical summary scales that are available in the interRAI mental health suite and **Table 4** shows the internal consistency results for seven parallel form scales from four interRAI mental health instruments in three countries. The scales include symptoms typically of interest in mental health settings; however, the two measures of functional status (activities of daily living (ADL) and instrumental activities of daily living (IADL)) are relevant to understanding disability in the assessed populations. Virtually all scales in all settings examined in **Table 4** meet or exceed the standard alpha value of 0.70 for good internal consistency and, in several instances, exceed 0.80 indicating excellent reliability. Good reliability is evident in both acute and longer-term service settings. In addition, the Positive Symptoms Scale, as assessed by police officers, has comparable reliability to that found when administered by mental health clinicians. The two exceptions in performance are the Aggressive Behavior Scale (ABS) in US community mental health settings and the Mania Scale in Canadian ESP settings. The US findings may be a function of an attenuated distribution of the ABS in the US pilot site (see **Table 5**). Site specific analyses of the Mania scale performance indicate that the problem was with one ESP pilot site, suggesting that training may have been a concern. Put differently, 39 of 41 evaluations of scale reliability in diverse mental health settings met or exceeded standard cut-points for good reliability.

**Table 3:** Summary of scales and algorithms in interRAI mental health instruments.

interRAI scale	Domain	Type of scale	Scale components	Range	Included in
Aggressive Behavior Scale	Aggressive behavior	Parallel form Sum of items	Verbal abuse; Physical abuse; Socially inappropriate/disruptive; Resists care	0–8	MH, CMH, ESP
Activities of Daily Living (ADL) Scale	Basic physical function	Parallel form Sum of items	Personal hygiene; Locomotion; Toilet use; Eating	0 to 16	MH, CMH, ESP
Negative Symptoms Scale	Negative symptoms	Parallel form Sum of items	Anhedonia;Withdrawal from activities of interest; Lack of motivation; Reduced social interactions	0 to 12	MH, CMH, ESP
CAGE-Crosswalk	Behavioral signs of addiction	Parallel form Sum of items	Need to cut down substance use; Angered by criticisms from others; Guilt; Drinking/using in am	0 to 4	MH, CMH
Cognitive Performance Scale	Cognitive function	Decision tree	Daily decision making; Short-term memory; Making self understood; Performance in eating	0 to 6	MH, CMH, ESP
Depressive Severity Index	Depressive symptoms	Parallel form Sum of items	Sad, pained facial expressions; Negative statements; Self-deprecation; Guilt/shame; Hopelessness	0 to 15	MH, CMH, ESP
Instrumental Activities of Daily Living Capacity	Higher level physical functioning	Parallel form Sum of items	Meal preparation; Ordinary housework; Managing finances; Managing medications; Phone use; Shopping; Transportation	0 to 30	MH, CMH, ESP
Mania	Mania symptoms	Parallel form Sum of items	Inflated self-worth; Hyperarousal; Irritability Increased sociability/hypersexuality; Pressured speech; Labile affect; Sleep problems—hypomania	0 to 20	MH, CMH, ESP
PAIN	Frequency and intensity of pain	Parallel form Sum of items	Pain frequency; Pain intensity	0 to 4	MH, CMH
Positive Symptoms Scale	Positive symptoms	Parallel form Sum of items	Hallucinations; Command hallucinations; Delusions; Abnormal thought process	0 to 12	MH, CMH, ESP,BMHS
Risk of Harm to Others	Harm to others	Decision tree	Violence/Extreme Behavior; Violent Ideation; Intimidation/threats; Aggressive Behavior Scale; Positive Symptoms Scale; Insight; Delusions; Sleep	0 to 6	MH, CMH, ESP
Self-Care Index	Self-care	Decision tree	Cognition; Positive Symptoms; Insight; Decreased Energy; Expressive Communication; Hygiene; Mania Scale; Anhedonia	0 to 6	MH, CMH, ESP
Severity of Self-harm Scale	Self-harm	Decision tree	Self-harm ideation; Suicide attempts; Suicide plan; Others concerned; Depressive severity scale; Positive Symptoms Scale; Cognitive Performance Scale	0 to 6	MH, CMH, ESP

**Table 4:** Internal consistency of clinical scales derived from interRAI Mental Health Instruments, by country.

Parallel Form Scale	Cronbach’s Alpha					
	CMH			BMHS Canada (n = 72,734)	ESP Canada (n = 5,249)	MH Canada (n = 230,790)
	Canada (n = 8,667)	Finland (n = 1,506)	New York (n = 2,689)			
Depressive Severity Index (0–15)	0.89	0.84	.84	NA^5^	.71	.75
Positive Symptoms Scale (0–12)	0.72	0.73	.74	.73	.72	.71
Negative Symptoms Scale (0–12)	0.90	0.84	.87	NA	.86	.85
Mania Scale (0–20)	0.70	0.68	.70	NA	.61	.77
Aggressive Behavior Scale (0–12)	0.70	0.71	.60	NA	.70	.77
Activities of Daily Living–Short Form (0–16)	0.81	0.74	.83	NA	NA	.89
Instrumental Activities of Daily Living Summary (0–30)	0.85	0.89	.79	NA	NA	.94

^5^NA—Scale not used in instrument.

**Table 5:** Univariate distributions of selected clinical scales derived from interRAI instruments by country.

Scale	CMH			ESP Canada (n = 5,249)	MH Canada (n = 230,790)
	Canada (n = 8,667)	Finland (n = 1,506)	US (NY state) (n = 2,689)		
	Percentage (n)				
Depressive Severity Index 0 1–3 4–7 8–15	31.0 (315) 30.5 (310) 20.8 (211) 17.6 (179)	13.6 (205) 27.3 (411) 27.4 (412) 31.7 (478)	47.4 (542) 26.4 (302) 15.0 (172) 11.2 (128)	24.3 (1287) 30.0 (1, 590) 19.5 (1, 032) 26.2 (1, 390)	25.0 (57, 631) 32.0 (73, 767) 25.9 (59.850) 17.1 (39, 542)
Mania Scale 0 1–3 4–8 9–20	27.4 (278) 41.3 (418) 23.6 (239) 7.7 (78)	13.4 (201) 32.2 (485) 37.6 (566) 16.9 (254)	54.0 (615) 29.0 (331) 13.3 (151) 3.8 (43)	31.8 (1, 638) 32.4 (1, 665) 25.0 (1, 285) 10.8 (558)	47.3 (109, 149) 25.7 (59, 333) 19.1 (43, 983) 7.9 (18, 325)
Aggressive Behavior Scale 0 1–3 4–6 7–12	65.4 (664) 27.6 (280) 6.0 (61) 1.1 (11)	61.7 (929) 29.3 (441) 7.2 (108) 1.9 (28)	77.8 (882) 18.9 (214) 2.7 (30) 0.7 (8)	81.8 (4, 293) 15.1 (794) 2.8 (145) 0.4 (18)	75.7 (174, 607) 13.4 (31, 014) 7.1 (16, 385) 3.8 (8, 784)
Cognitive Performance Scale 0 1–2 3–6	46.7 (474) 47.2 (480) 6.1 (62)	33.7 (507) 54.8 (825) 11.6 (174)	59.3 (665) 36.8 (413) 3.9 (44)	81.2 (4251) 15.9 (834) 2.9 (150)	67.1 (154, 827) 24.1 (55, 686) 8.8 (20, 277)
Instrumental Activities of Daily Living Summary Scale 0 1–3 4–9 10–18 19–30	50.1 (495) 13.3 (131) 17.0 (168) 10.9 (108) 8.8 (87)	39.2 (590) 10.6 (160) 14.3 (216)) 19.2 (289) 16.7 (251)	49.4 (580) 15.7 (185) 19.7 (232) 10.9 (128) 4.3 (50)	NA^6^	63.4 (146, 298) 11.4 (26, 276) 10.0 (23, 099) 6.8 (15, 629) 8.4 (19, 488)

^6^IADL Scale not collected in interRAI ESP.

#### Validity

Validity is a more complex psychometric issue than reliability, requiring evaluation through a number of methods. The key questions of interest include: does this item or scale measure what I think it measures? Does the assessment address the important characteristics affecting the person's well-being? How does the instrument compare with other widely used systems? Do risk indicators actually predict future outcomes of interest?

##### Face and Content Validity

Face and content validity are necessary, but not sufficient criteria to meet for developing an assessment system. The interRAI development process typically addresses these through extensive involvement of front-line practitioners and researchers combined with reviews of the current literature. For example, as part of the development effort for the care planning protocols, key informants from several nations were asked: a) How consistent is the MH-CAP with the Recovery Model as used by your organization? (83% rated the protocol reviewed to be mostly or completely consistent); b) How consistent is the MH CAP with guidelines/best practices used by your organization? (92% rated them as mostly or completely consistent); and c) How would you rate this CAP in terms of its use as a support for care planning in this area? (90% rated them as good or excellent). In addition, critical feedback from informants was used to inform final revisions to the penultimate versions of these care planning protocols.

##### Convergent Validity

The patterns of associations in a dataset can provide insights about whether the instrument measures what one thinks it measures. The initial version of the MH was successfully evaluated by examining the presence of expected associations with age of cognitive loss and functional decline, the relationship between depression measures in suicide related indicators, readmission rates and problems with medication management, and cognitive performance with behavior (248).

As an extension of this approach, one might examine the extent to which hallmark clinical symptoms of various psychiatric diagnoses are associated with the presence of those diagnoses in the assessment data. A clear positive association would provide reassurance that the instrument measures symptoms that have meaning to clinicians. **Table 6** shows the relationship that could be expected between various mental status indicators and provisional diagnoses of neurocognitive disorders; substance related and addictive disorders; schizophrenia spectrum and other psychotic disorders; and depressive disorders. For the first three diagnoses in all three settings, the anticipated relationships with symptoms are strong, in the appropriate directions, and the c-statistics are generally at the 0.80 level or higher. For depression diagnoses, the odds ratios for the depressive severity index and social withdrawal index are in the appropriate directions, but the associations with depressive symptoms are stronger than with social withdrawal (which may relate to other diagnoses). The c-statistics for depression diagnoses are between 0.64 and 0.70.

**Table 6:** Odds ratios (95% CL) for provisional psychiatric diagnoses by associated symptoms and setting, Canada.

Provisional diagnosis	Covariate	ESP (n = 5, 235)		CMH (n = 11, 641)		MH (n = 230, 790)	
		Odds Ratio	c	Odds Ratio	c	Odds Ratio	c
Neurocognitive disorders	Cognitive Performance Scale (ref = 0) 1–2 3–6	12.73 (7.77–20.86) 47.89 (27.10–84.62)	.82	10.06 (6.73–15.04) 88.93 (55.68–142.05)	.82	2.90 (2.74–3.08) 8.57 (8.01–9.16)	.86
Substance related & addictive disorders	Misuse prescription meds (ref = no) Count of current substances used Days drank to intoxication (ref = 0) 1–8 9-daily 5+ drinks in single sitting (ref = 0–4) CAGE crosswalk score (ref = 0) 1 2 3 4	2.07 (1.65–2.61) 2.37 (2.16–2.59) 2.49 (2.07–2.99) 6.77 (5.40–8.49) NA NA	.79	(0.86–1.55) 1.42 (1.12–1.81) NA 1.54 (1.34–1.77) 5.25 (4.06–6.78) 8.88 (6.62–11.91) 16.78 (12.27–22.94) 19.49 (13.40–28.34)	.78	1.24 (1.20–1.28) 2.28 (2.24–2.32) NA 2.65 (2.56–2.73) 4.02 (3.86–4.19) 7.14 (6.85–7.45) 14.80 (15.17–15.45) 26.69 (25.36–28.08)	.87
Schizophrenia spectrum & other psychotic disorders	Positive Symptoms Scale (ref = 0) 1–2 3–5 6–12 Insight to MH condition (ref = full) Partial None	3.11 (1.87–5.17) 8.73 (6.68–11.42) 21.58 (16.97–27.44) 1.72 (1.39–2.14) 1.63 (1.21–2.20)	.84	3.42 (2.95–3.97) 4.20 (3.54–4.98) 5.64 (4.20–7.57) 2.18 (1.95–2.44) 2.81 (2.16–3.64)	.71	4.10 (3.97–4.24) 6.96 (6.77–7.16) 14.77 (14.34–15.22) 1.79 (1.74–1.85) 2.26 (2.17–2.34)	.80
Depressive disorders	Depressive Severity Index (ref = 0) 1–3 4–7 8–15 Social Withdrawal Scale (ref = 0) 1–4 5–8 9–12	1.35 (1.15–1.59) 1.74 (1.45–2.10) 2.13 (1.77–2.57) 1.01 (0.86–1.19) 1.38 (1.16–1.65) 2.30 (1.95–2.72)	.64	2.20 (1.86–2.60) 4.09 (3.25–5.16) 6.25 (4.55–8.59) 1.66 (1.40–1.96) 1.57 (1.24–1.98) 1.41 (1.05–1.90)	.70	1.49 (1.46–1.53) 2.34 (2.29–2.40) 3.78 (3.67–3.90) 1.14 (1.12–1.17) 1.41 (1.38–1.45) 1.60 (1.55–1.64)	.65

##### Criterion *Validity*

A common approach to validation is to compare a new instrument to another scale that is recognized as a “gold standard” measure. The challenge in mental health is that few measures are universally accepted as a gold standard, and biological markers are not particularly useful as they might be in somatic illness. Previous interRAI research has established the criterion validity of the following scales and items: pain scale *vs* Visual Analogue Scale (254); Cognitive Performance Scale versus Mini Mental State Examination and Montreal Cognitive Assessment (255, 256); Depression Rating Scale versus Hamilton and Cornell Scales and psychiatrists ratings (257); Aggressive Behavior Scale versus Cohen Mansfield Agitation Inventory (203); delirium algorithms versus the Confusion Assessment Method (258); recorded diagnoses versus acute hospital medical records (259); and mental health care planning triggers versus clinician judgement (260). In a developmental project to refine an earlier version of the interRAI MH, criterion validity studies with 876 patients in 11 psychiatric hospitals/units yielded the following correlations: Aggressive Behavior Scale with Neuropsychiatric Inventory Total Score r = 0.50 and with PANSS Aggression Risk Profile r = 0.58; Pain Scale with McGill Pain Questionnaire r = 0.64; Positive Symptoms Scale with PANSS Positive Symptoms r = 0.62; Negative Symptoms Scale with PANSS Negative Symptoms r = 0.49 (261).

##### Predictive Validity

Arguably the most important (and difficult) form of validity to establish for an assessment system is predictive validity. Presumably, the ultimate purpose of assessment is to guide interventions that will have an impact on a future clinical trajectory of change. This approach was used extensively to identify triggering rules for interRAI's mental health care planning protocols (see discussion below). Examples of publications reporting on this type of validity include studies of inpatient violence (262), re-hospitalization (82); and suicide behaviors (196, 197).

### Applications of interRAI Instruments

Critically, all interRAI assessment instruments are designed to be used as part of normal clinical practice to serve multiple functions for multiple audiences (263), including: a) care planning; b) outcome measurement; c) resource allocation; and d) quality improvement. In addition, the aggregated data compiled as part of regular use can be employed for policy analysis, evaluation and research. Examples of peer-reviewed health services research based on interRAI MH data include: mental health needs of prisoners (264, 265); mental health service planning (266); access to psychiatrists by linguistic minorities (267) and nursing home residents (268); use of ECT by psychiatric inpatients (269); prolonged stays (270, 271); length of stay (272, 273); and restrictions in movement in forensic patients (172). Examples of clinically oriented research with these data include studies of: sexual dysfunction (274, 275); incontinence (276, 277); discharges against medical advice (229, 278); medication non-adherence and misuse (229, 279, 280); restraint and acute control medication use (281); and pharmacoepidemiological studies (282).

#### Care Planning

The Clinical Assessment Protocols (CAPs) associated with the suite of interRAI assessments are care planning guidelines designed with a common structure and clinical approach, but they are adapted to the populations and service settings for which they should be used (175, 192, 283–286). Each CAP contains five main components: a) a description of the importance of the clinical issue; b) goals of care underlying CAP design; c) triggering algorithms that use the assessment items to classify persons for different care planning approaches; d) guidelines to consider for both immediate actions to deal with safety concerns and longer-term strategies that may be used; and e) additional reference materials and resources that may be consulted.

The CAP development process involves three main approaches. First, interRAI's large international network is deployed to engage with clinicians, researchers, policy makers, and advocacy groups to discuss priorities and approaches to care recommended in different countries. Second, reviews of literature and best practice guidelines help identify practical, evidence-based interventions for specific clinical concerns. Third, interRAI's large longitudinal data holdings are used to develop and validate the predictive validity of CAP “triggering” algorithms. The results of these analyses are included in the mental health CAP descriptions to identify expected triggering rates and outcomes for inpatient, community and emergency settings. Finally, agreement with clinician ratings was also used to validate and refine the CAP triggering rules (287).

CAPs are designed using several key principles. The intervention guidelines must be rooted in empirical evidence from the peer reviewed literature in multiple continents so as not to reflect only one system of care. In addition, all mental health CAPs are framed on recovery principles (108, 111), including shared decision-making and support of the person's autonomy calibrated to his/her current level of functioning. The approach engages the person and, where appropriate, the informal support network in collaborative discussion to review the assessment findings about the person's strengths and needs and to identify the person's preferences for how CAP issues will be addressed, if at all. CAPs are not intended to be robotic care planning libraries. Rather, they provide qualitative “interview guides” that outline potential responses to the quantitatively defined triggering algorithms derived from the standardized assessment. Similarly, the CAPs are not a diagnostic system; they are designed to focus on intervention strategies at the person, family, and community levels that might enhance the person's quality of life in as many dimensions as possible.

**Figure 2:**
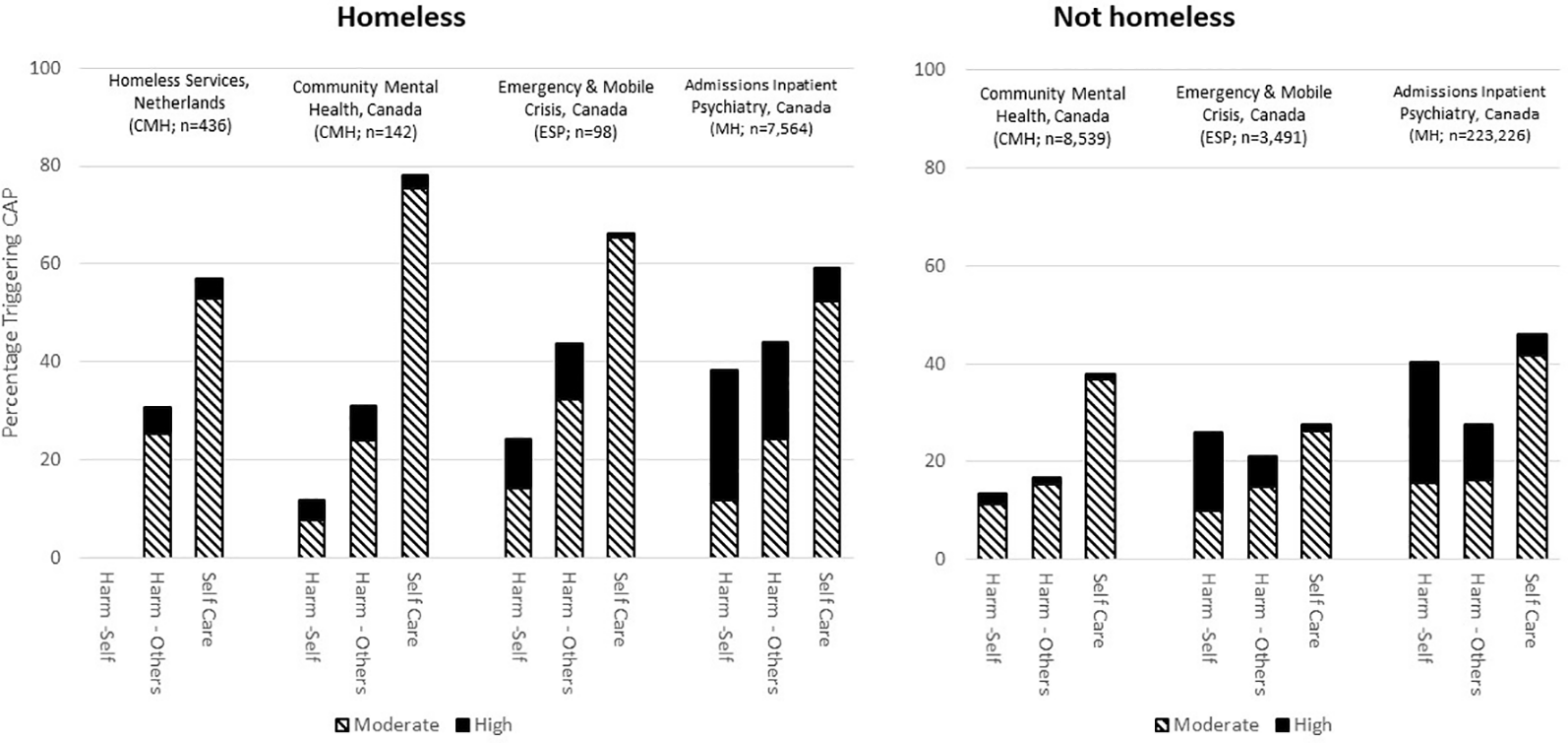
Triggering rates for three patient safety CAPs, by homelessness, setting and country.

The CAPs triggered by the interRAI mental health suite are listed in **Table 7**. The interRAI MH and CMH trigger all CAPs, but have somewhat different protocols for informal supports. The interRAI ESP triggers mainly the patient safety related CAPs as priority issues for crisis situations. Previous publications have highlighted triggering patterns and outcomes associated with the CAPs for traumatic life events (288), education and employment (289), and harm to others (262). **Figure 2** shows the triggering rates for patient safety CAPs by homelessness in different service settings. The triggering rates for these CAPs in homeless persons in four settings (community dwelling recipients of Dutch homeless services, Canadian community mental health, emergency/mobile crisis, and inpatient settings) are lowest for the purposeful self-harm CAP and highest for the self-care CAP. With the transition from community to acute hospital-based services, the triggering rates for purposeful self-harm and harm to others are higher for all groups. Conversely, self-care triggers at the highest rate for homeless persons in community mental health settings. For non-homeless populations, the triggering rates for patient safety issues are generally lower than with homeless persons, but the rates and severity levels are also higher in emergency and hospital settings than in the community.

**Table 7:** List of clinical assessment protocols triggered by different interrai mental health instruments.

CAP Name	Trigger Levels (Number and focus)	interRAI Mental Health Assessment		
		Inpatient (MH)	Community (CMH)	Emergency (ESP)
**SAFETY** Harm to Others Suicidality and Purposeful Self-Harm Self-Care	2 levels; risk severity 2 levels; risk severity 2 levels; risk severity	✓ ✓ ✓	✓ ✓ ✓	✓ ✓ ✓
**SOCIAL LIFE** Social Relationships Informal Support Support Systems for Discharge Interpersonal Conflict Traumatic Life Events Criminal Activity	2 levels; degree of isolation, family dysfunction 2 levels; type of support needed 1 level; availability of support on discharge 2 levels; extent of conflict 2 levels; immediate safety, ongoing impact 1 level; violent or non-violent criminal behavior	✓ × ✓ ✓ ✓ ✓	✓ ✓ × ✓ ✓ ✓	× × × × × ×
**ECONOMIC ISSUES** Personal Finances Education and Employment	2 levels; economic hardship; IADL capacity 2 levels; reduce risk, support employment/education	✓ ✓	✓ ✓	× ×
**AUTONOMY** Control Interventions Medication Management and Adherence Rehospitalization	2 levels; use in emergency and non-emergency situations 2 levels; IADL & adherence, stopped meds/side effects 2 levels; risk severity	✓ ✓ ✓	✓ ✓ ✓	× × ×
**HEALTH PROMOTION** Smoking Substance Use Weight Management Exercise Sleep Disturbance Pain Falls	2 levels; withdrawal symptoms, encourage cessation 2 levels; current problematic use, prior problematic use 2 levels; problem BMI; problematic eating behaviors 2 levels; physical activity & presence of health concerns 2 levels; sleep disturbance & cognitive impairment 2 levels; treatment priority level 2 levels; risk severity	✓ ✓ ✓ ✓ ✓ ✓ ✓	✓ ✓ ✓ ✓ ✓ ✓ ✓	✓ × × × × × ×

**Figure 3:**
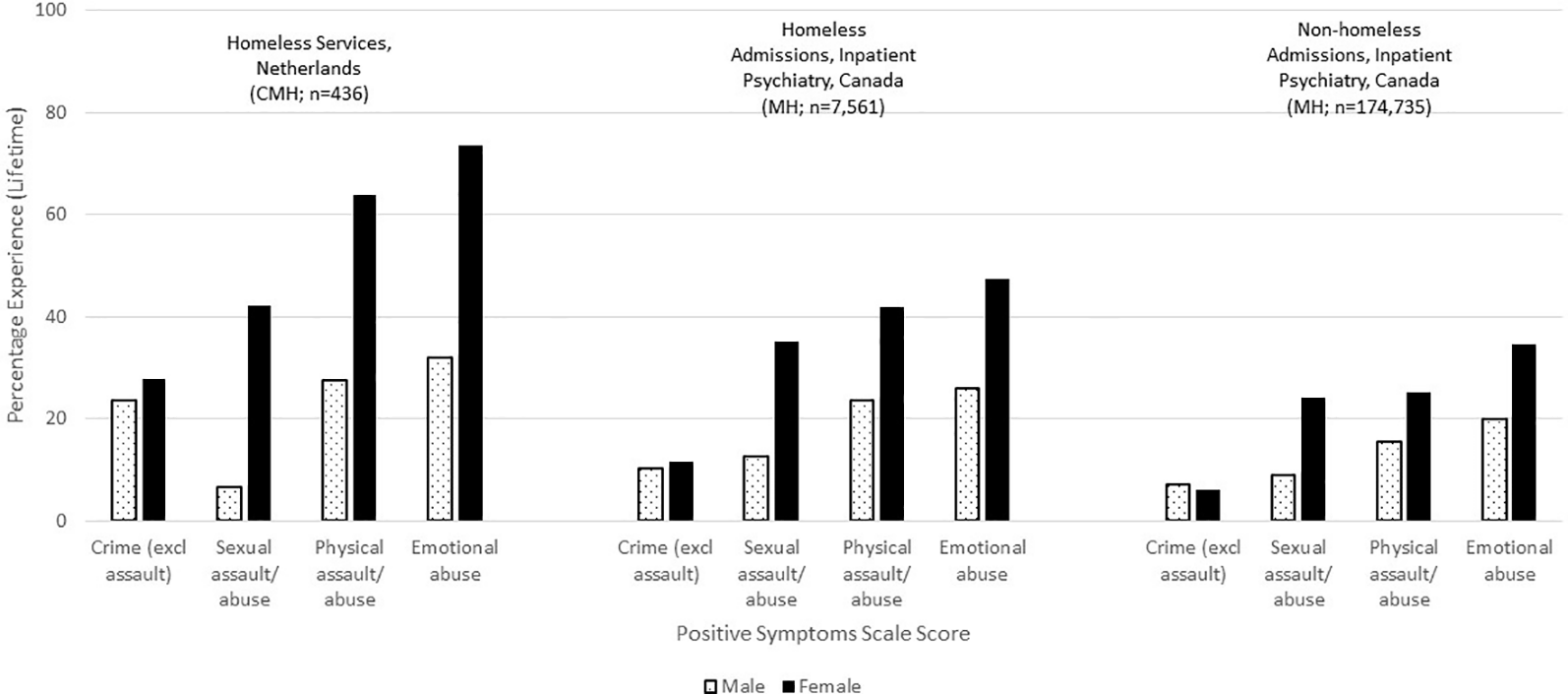
Percentage distributions of lifetime history of victimization by homelessness and service setting, Canada and Netherlands.

The CAPs can be used at the person-level to inform care planning, or aggregated at the organization or population levels to provide estimates for need analyses in various domains. The CAPs focus on current issues that increase the risk of adverse outcomes or those that might be addressed to support recovery. For example, the CAP for traumatic life events (288) targets two sub-groups for intervention: a) persons who face immediate threats to their safety due to assaults, abuse or criminal victimization that has occurred within the last 7 days; and b) persons who experienced potentially traumatic life events and who described those events as inducing a sense of intense fear or horror. The latter group were found to have elevated rates of PTSD related symptoms (288). However, from a population perspective it is also sometimes useful to examine patterns of lifetime exposure to adverse circumstances to inform policy development. **Figure 3** shows the lifetime rates of criminal victimization (excluding assaults, abuse), and three types of assault or abuse by homelessness and gender. Whether considering the homeless population receiving services in the community or psychiatric inpatients admitted from homeless settings, the lifetime rates of criminal victimization, assault or abuse are higher than in the non-homeless population. Moreover, women (whether homeless or not) are systematically more likely to have experienced the three types of abuse in their lives.

#### Outcome Measurement

interRAI instruments contain numerous embedded scales (see **Tables 3** and **5**) and individual items that summarize the presence/absence and severity of needs at a given point in time. These may be examined longitudinally with reassessments or when linked to previous interRAI assessments done in other care settings. At the person-level, these longitudinal changes provide insights about the person's changes in strengths and needs, response to treatment, and progression or recovery from illness.

**Figure 4:**
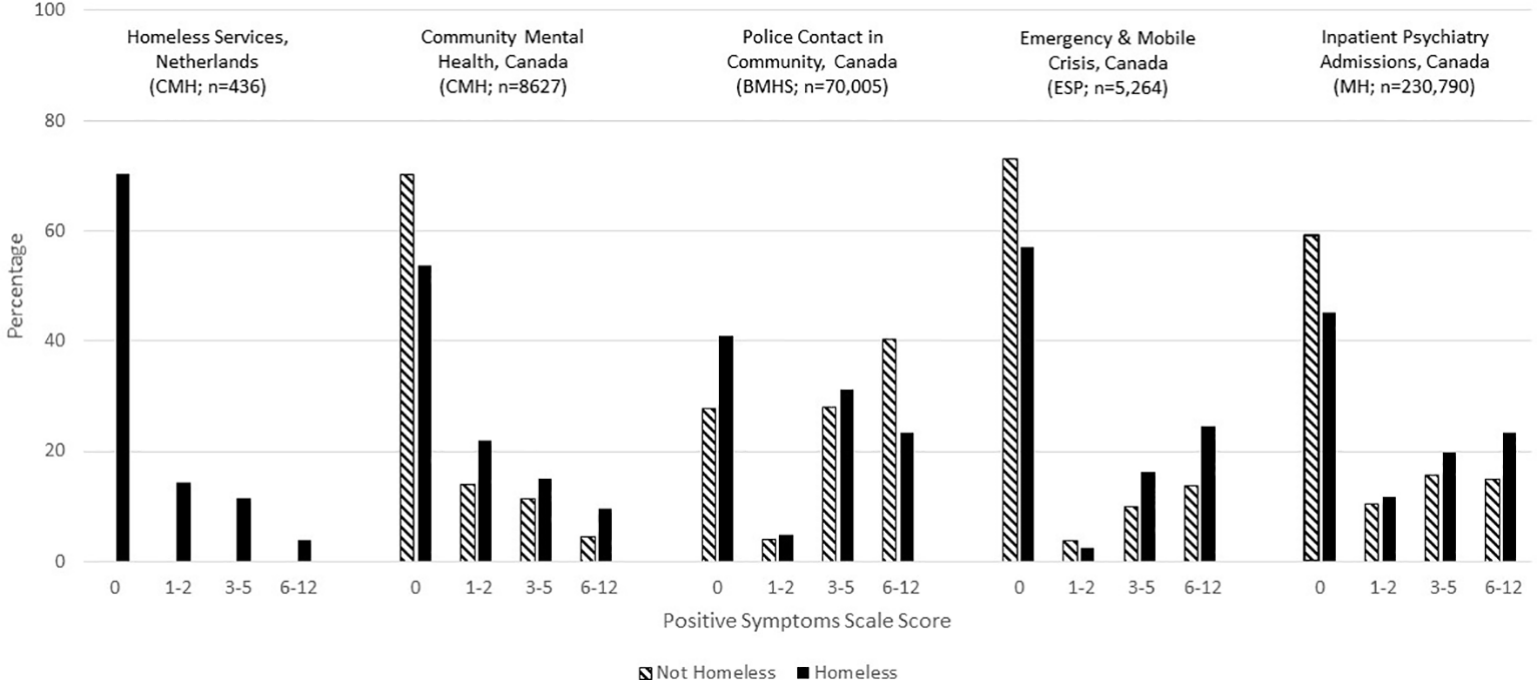
Percentage distributions of positive symptoms scale score by homelessness and service setting, Canada and Netherlands.

**Figure 4** shows the distribution of the Positive Symptoms Scale (see **Table 3** for scale description) for homeless and non-homeless persons in five care settings. About 30% of persons in homeless services in the Netherlands, who receive only limited mental health supports, have signs of psychosis compared with almost half of the homeless persons in Canadian community mental health agencies. Severity of positive symptoms increases among those in contact with hospital or emergency mental health services, but it is most pronounced for homeless persons at the time of contact with police. In all settings, except police contacts, the severity of positive symptoms is greater for homeless than non-homeless persons.

**Figure 5:**
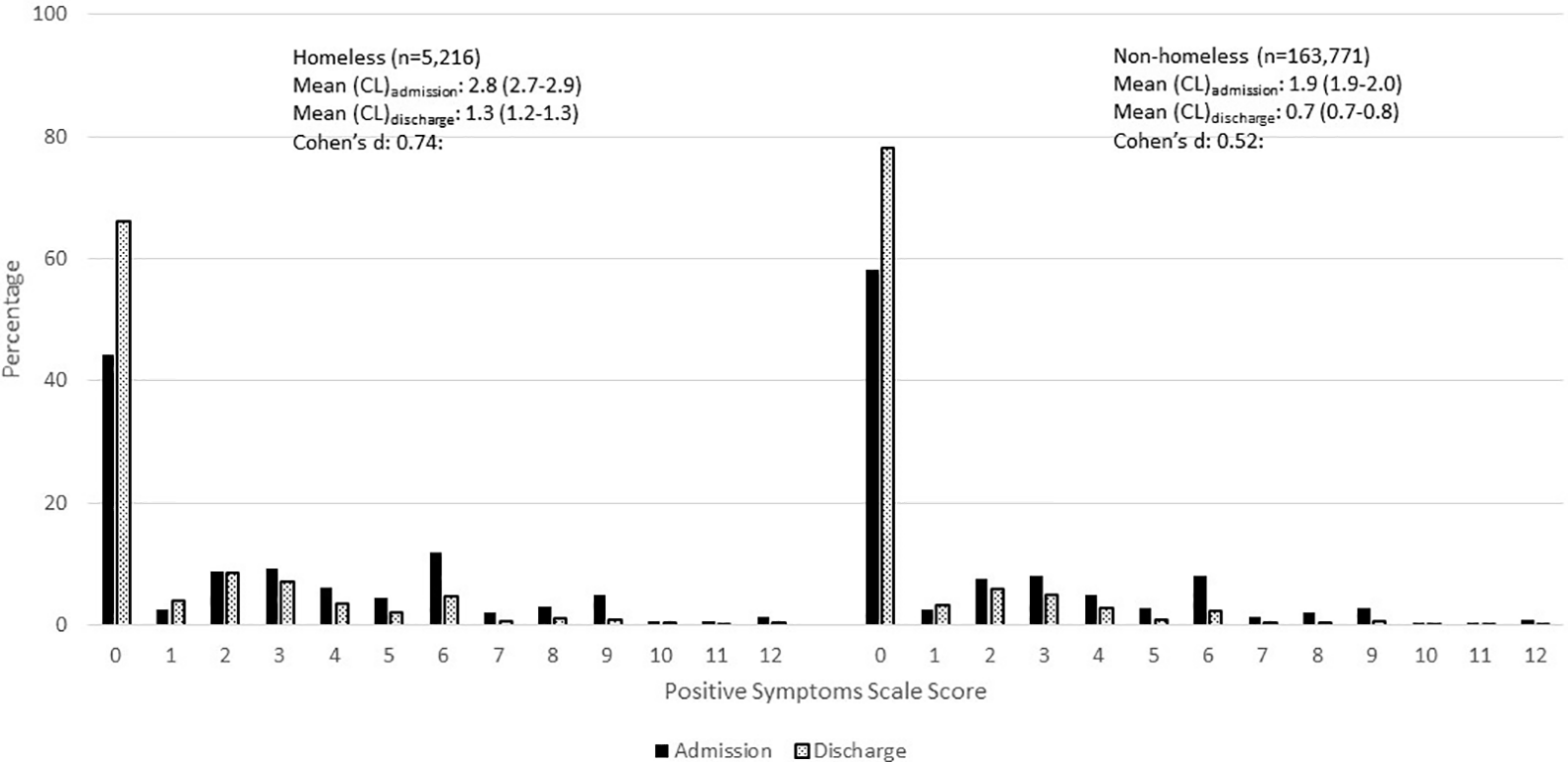
Percentage distribution of positive symptoms scale scores at admission and discharge within 90 days, inpatient psychiatry, Canada.

**Figure 5** shows the transitions in the Positive Symptoms Scores by homelessness from the time of admission to discharge for persons with stays of less than 90 days. Both populations improved substantially in symptom severity, but the scores were worse at admission and discharge for homeless persons. 

#### Quality Indicators

interRAI has an extensive history of developing risk-adjusted outcome-based quality indicators. While scales and items tracked over time for individuals can be useful for person-level care planning, the longitudinal data may be aggregated at the population level to benchmark performance for internal quality improvement, accreditation and public reporting (290–297). Although interRAI's nursing home quality indicators are reported on-line nationally (298, 299), its mental health quality indicators (MHQIs) are currently provided to hospitals for internal use only. A detailed summary of these risk-adjusted quality indicators is provided elsewhere (300). The majority of these indicators are outcome-based, with the exception of a limited number of process indicators dealing with restraint and acute control medication use. There are two main outcomes: a) improvement in symptoms for persons who have non-zero values for the scale of interest at admission; b) failure to improve or worsening for persons who do not have maximum scores at admission. The outcomes include common psychiatric symptoms (*e.g.*, depressive symptoms), functional indicators (*e.g.*, medication management), and others that are less commonly tracked (*e.g.*, pain).

**Figure 6:**
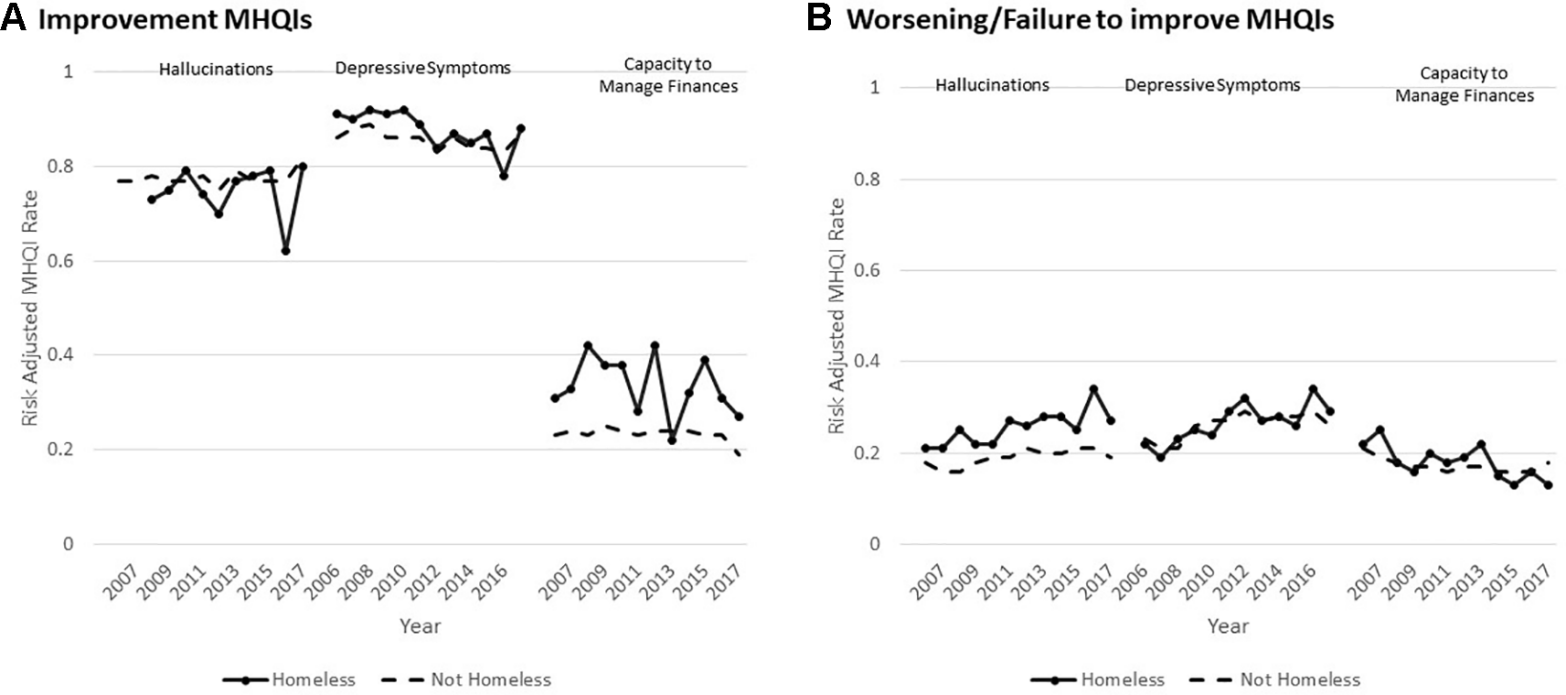
Temporal changes in three risk adjusted mental health quality indicators by year, inpatient psychiatry, Canada. **Figure 6A** shows the risk adjusted rates of improvement in hallucinations, depressive symptoms, and capacity to manage finances in the last 90 days or at discharge (if discharged less than 90 days since baseline assessment). **Figure 6B** shows the risk adjusted rates of worsening of or failure to improve in hallucinations, depressive symptoms, and capacity to manage finances in the last 90 days or at discharge (if discharged less than 90 days since baseline assessment).

**Figure 6A** shows the population-based risk-adjusted MHQI rates over time for three improvement indicators stratified by homelessness (homeless *vs* not homeless) in Canadian inpatient psychiatry. The rates are more volatile for the homeless indicators due to smaller sample sizes, but there are only small differences between the two subgroups for improvement in hallucinations and depressive symptoms. Both of these indicators show improvement rates to be above 0.70 as the risk-adjusted proportion improving from baseline to follow-up. On the other hand, for the homeless group, improvement rates are much lower for capacity to manage finances, despite this group having a somewhat better improvement rate than non-homeless persons.

**Figure 6B** shows three risk-adjusted MHQIs for worsening or failure to improve in the same three domains. Rates of worsening or failure to improve increased (indicating poorer care) over time with notably higher rates for homeless persons. The indicator for depressive symptoms also showed poorer performance over time, but no substantive difference between subpopulations. On the other hand, rates of worsening or failure to improve in managing finances declined over time for both groups.

#### Resource Allocation

interRAI systems may also be used to inform decisions about the allocation of health care resources at the person and population levels. In non-mental health settings, interRAI systems have been developed to prioritize access to community and institutional services (301) and for eligibility determination in long-term care (302). Mental health counterparts to this work include a level of care framework based on the interRAI MH (303), analyses of service complexity for children with mental health needs and developmental disability based on the ChYMH-DD (304), and a decision-support algorithm describing resource intensity based on the interRAI ChYMH (305).

Case-mix systems have considerable value for informing a variety of health care decisions, including funding methodologies (306). These systems tend to have two main components: a) a classification system, based on clinical characteristics, to group service users into categories with similar levels of resource use; and b) a weighting system (case-mix indexes or CMIs) assigns ratio-level numeric values to these groups that can, among other functions, be applied to funding formulas in payment systems. interRAI-based case-mix systems are available for nursing homes (307–309), home care (310), intellectual disability services (311, 312), and intervener/interpreter services for dual sensory loss (313).

The Diagnosis Related Groups (314) system is widely used in acute general hospitals, but a consensus was reached over three decades ago that the system was inadequate for describing resource use in psychiatry (315). A number of studies pointed to the potential to use per-diem based case-mix systems that estimate costs of care per day of stay, rather than episode-based systems that attempt to predict length of stay (316–318). Most research of this type in mental health has been in hospital settings with only modest progress in community mental health services (319). In addition, although better than episode-based models, the ability to explain variance in resource use in psychiatry is lower than in more homogeneous care settings such as nursing homes (320).

**Figure 7:**
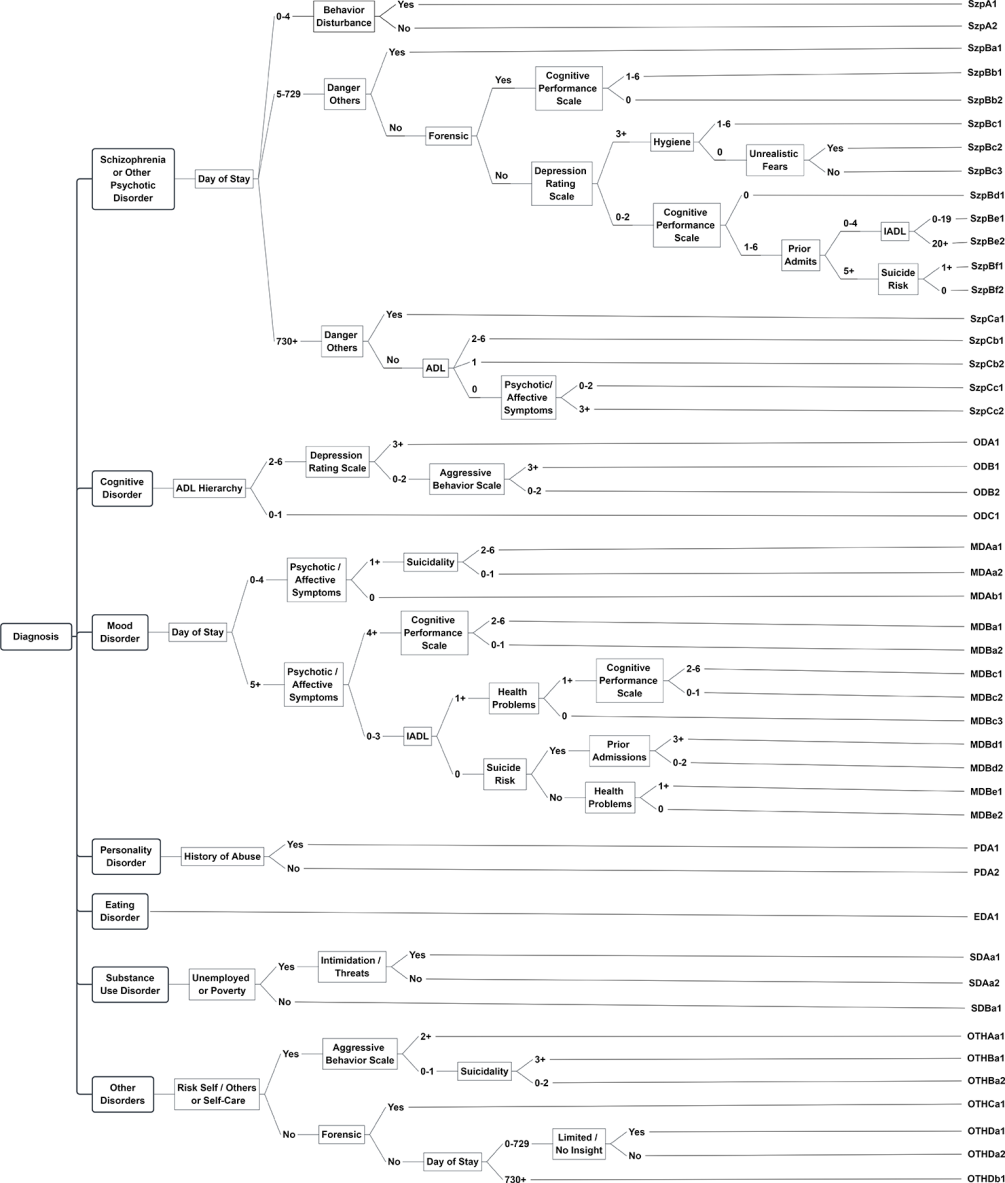
System for classification of inpatient psychiatry (SCIPP) schematic. Blue boxes represent decision points, gold ovals represent terminal SCIPP groups.

The System for Classification of In-Patient Psychiatry (SCIPP) is a per-diem case-mix system for inpatient mental health services based on an earlier version of the interRAI MH (321–324). SCIPP was developed through a staff time measurement study of about 2,000 patients in 34 psychiatric hospitals in three Canadian provinces. The System for Classification of In-Patient Psychiatry (SCIPP) includes about 100 variables for a 47-group algorithm (see **Figure 7**) explaining about 26% of variance in per diem resource use among adult psychiatric patients. There is an 8.4 to 1 range in CMIs across the SCIPP groups. Careful attention was paid to avoiding the use of service variables, facility variables, gameable items and items that had poor psychometric properties. The SCIPP algorithm provides an important step forward in case-mix research for psychiatry. It achieves a higher explained variance than has been possible in episode-based systems, and does so without the use of independent variables that would be problematic to administer as part of a prospective payment system.

**Figure 8:**
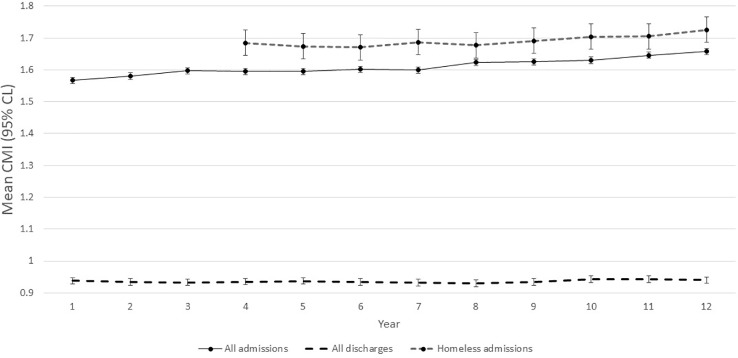
Trends in mean in (95% CL) SCIPP case-mix index values, inpatient psychiatry, Canada.

**Figure 8** shows historical trends in the mean CMI scores in Canadian psychiatric hospitals/units using the MH as part of routine practice. Between 2006 and 2017, the mean admission CMIs rose from 1.567 to 1.657 equating to a 5.7% increase in resource intensity. On the other hand, the discharge CMIs were virtually unchanged over that time period at about 0.940. This indicates two main points: a) there is roughly a 55% drop in resource intensity from admission to discharge associated with the alleviation of symptoms related to mental health and co-morbid conditions; and b) hospitals admitted heavier patients over time without changes in their resource intensity when discharged. In addition, homeless persons were consistently more resource intensive at admission compared with the general populations with relative differences ranging between 5.6 and 4.2%. 

## Future Directions

Although much has been accomplished through interRAI's two decades of research on its mental health instruments, some limitations of the existing instruments must be addressed in order to continue to improve their utility.

There is a clear need for more cross-national research and implementation of the mental health suite. Low-middle income nations are of particular interest, but adaptations to lower resource environments are likely to be necessary. Many such countries are undertaking dramatic reforms of their mental health systems to place greater emphasis on decentralized networks and primary care (325–328) where interRAI's mental health systems have not yet been used. Another gap is the lack of a triage and screening tool for intake services and public “help” lines. These gaps may be addressed, in part, through the development of companion self-report instruments to reach populations not receiving mental health services. Such instruments might also be useful for settings with few available mental health professionals.

Another limitation is that the translation of interRAI instruments into normal clinical practice is heavily dependent on robust implementation strategies led by highly committed mental health professionals. The ability to realize the full potential of all clinical and management applications can be hindered by implementations that emphasize the data collection aspects of the system rather than its clinical use (329, 330). Moreover, even though interRAI instruments are designed to function as an integrated, cross-sectoral system, the sharing of assessment results between clinicians may not occur for reasons including ineffective communication and collaboration between those sectors (160). Further, relatively little work has been done to date on how best to present interRAI assessment results to patients as part of the shared decision-making process.

Also, while a great deal of validation work has been done, there is need for additional research on criterion validity related to mania, anxiety, and trauma. Predictive validity studies related to suicide and forensic risk are underway. It would be useful to complete inter-rater reliability studies between assessments completed by nurses or social workers and those done by clinical psychologists and psychiatrists. Of particular importance is the need for validation and refinement studies for the SCIPP system, particularly for specialized populations (*e.g.*, forensics). Also, there is no case-mix system available at this stage for the community mental health instrument.

The availability of rich longitudinal datasets with over 1 million observations of over 400 clinical variables creates opportunities for applying new machine learning and artificial intelligence tools. When combined with real-time analytic capabilities in electronic medical records, the potential to create new personalized medicine applications that place the person's data in the context of population level data is considerable. In addition, linkage to other data sources—wearable devices, registry and administrative data, biomarker data (*e.g.*, laboratory values, genetic data)—provides great opportunity for novel insights and innovative improvements to mental health services. Such discoveries have already been made by linking Icelandic genetic data and interRAI nursing home data to examine Alzheimer disease and cognitive decline (331, 332).

Finally, if the next 20 years of use of interRAI mental health systems sees the same degree of growth in its child/youth instruments, the future opportunities for understanding mental health from a life course perspective can be realized. The availability of scientifically sound, standardized, and fully compatible measures that follow persons living with mental illness from the earliest life stages throughout adulthood will be an unprecedented new opportunity to develop solutions to alleviate the impact of mental illness for persons of all ages.

## Author’s Note

The authors are all part of the interRAI network.

## Ethics Statement

Ethics clearance for secondary analyses of interRAI data gathered by other organizations was obtained from the University of Waterloo (ORE#30173).

## Author Contributions

All authors contributed to the formulation of the ideas presented in the study and provided critical feedback to the manuscript. JPH drafted the first version of the manuscript, with major sections contributed by CE, MF, JH, AH, LM, CP, SS and CA. JPH, JF, MJ, BF, and LM made editorial changes to initial drafts and in response to reviewers' comments. JPH, BF, and CE conducted data analyses of Canadian, US and Dutch data, respectively.

## Funding

Some aspects of this research were funded by the Health Transition Fund, Health Canada G03-05691. Research on homelessness in the Netherlands was commissioned and funded with support from various municipalities and human services organizations. Maastricht University and Radboud University, Nijmegen provide core financial support for the scientific use of data from these studies. Data from implementations in Canada, United States and Finland were made available based on data sharing agreements with interRAI. 

## Conflict of Interest

The authors declare that the research was conducted in the absence of any commercial or financial relationships that could be considered as a potential conflict of interest.
