# Supplementary material for: The interRAI Suite of Mental Health Assessment Instruments: An Integrated System for the Continuum of Care
Source: Front Psychiatry. 2020 Jan 17;10:926. doi: 10.3389/fpsyt.2019.00926 (PMC6978285; doi:10.3389/fpsyt.2019.00926)
Supplement: Supplementary file 1 [file Table_1.docx]

**Supplementary Table 1. Sample Characteristics, Community Mental Health Clients in Canada (Ontario and Newfoundland), Finland, and New York State**

| **Scale** | **CMH** | | | **BMHS**  **Canada**  **(n=72,734)** | **ESP**  **Canada**  **(n=5,249)** | **MH**  **Canada**  **(n=230,790)** |
| --- | --- | --- | --- | --- | --- | --- |
|  | **Canada (n=8,667)** | **Finland (n=1,506)** | **New York**  **(n=2,689)** |  |  |  |
|  | **Percentage (n)** | | | | | |
| *Age*  - 18-44  - 45-64  - 65+ | 44.4 (1749)  43.6 (1718)  11.9 (470) | 47.6 (716)  44.1 (664)  8.4 (126) | 35.9 (1029)  61.5 (1764)  2.6 (75) | 68.2 (50087)  24.0 (17627)  7.8 (5712) | 67.9 (1381)  26.2 (534)  5.9 (120) | 50.9 (114,976)  33.8 (76426)  15.4 (34717) |
| *Sex*  - male  - female | 48.2 (4189)  51.8 (4499) | 48.6 (731)  51.5 (775) | 48.7 (1393)  51.3 (1466) | 36.2 (26600)  63.8 (46826) | 48.4 (2538)  51.6 (2703) | 51.1 (117989)  48.9 (112730) |
| *Marital status*  - married  - other | 27.5 (2203)  72.5 (5818) | 16.7 (190)  83.3 (950) | 12.3 (351)  86.7 (2495) | NA | NA | 30.3 (69916)  69.7 (160874) |
| *Type of housing*  - private home/apt/room  - other | 86.3 (7394)  13.7 (1170) | 52.1 (588)  47.9 (541) | 71.2 (1986)  28.8 (804) | NA | 93.4 (4909)  6.6 (348) | 87.8 (160085)  12.2 (22263) |
| *Homeless*  - no  - yes | 98.3 (8625)  1.7 (148) | 82.0 (1235)  18.0 (271) | 100.0 (2867)  0.0 (1) | 89.1 (63171)  10.9 (7702) | 96.8 (5154)  3.2 (169) | 96.7 (223226)  3.3 (7564) |
| *Provisional diagnoses* - schizophrenia  - mood  - substance use  - cognitive  - anxiety  - personality | 33.2 (2915)  38.8 (3403)  10.2 (898)  2.8 (245)  26.1 (2287)  8.6 (751) | 52.5 (791)  40.5 (610)  10.0 (150)  0.0 (0)  10.1 (152)  12.1 (182) | 35.9 (1030)  47.1 (1351)  25.2 (723)  3.3 (95)  40.3 (1156)  9.3 (266) | NA | 12.3 (655)  42.0 (2233)  21.1 (1122)  2.1 (110)  27.7 (1473)  17.1 (910) | 27.4 (63248)  52.9 (122080)  27.0 (62304)  7.6 (17623)  15.4 (35508)  8.6 (19912) |
| *Intellectual disability*  - no  - yes | 95.9 (8418)  4.1 (355) | 96.3 (1450)  3.7 (156) | 94.5 (2711)  5.5 (157) | NA | NA | 96.3 (222178)  3.7 (8612) |
| *Lifetime inpatient admissions*  - None  - 1  - 2-4  - 5+ | 45.4 (3716)  32.9 (2691)  9.4 (765)  12.3 (1008) | 25.2 (379)  29.4 (443)  12.8 (193)  32.6 (491) | 31.7 (907)  32.2 (919)  12.5 (358)  23.6 (674) | NA | 63.8 (2735)  25.0 (1070)  6.0 (255)  5.3 (228) | 40.3 (93110)  38.8 (87211)  10.6 (24490)  11.3 (25979) |
